# S55746 is a novel orally active BCL-2 selective and potent inhibitor that impairs hematological tumor growth

**DOI:** 10.18632/oncotarget.24744

**Published:** 2018-04-13

**Authors:** Patrick Casara, James Davidson, Audrey Claperon, Gaëtane Le Toumelin-Braizat, Meike Vogler, Alain Bruno, Maïa Chanrion, Gaëlle Lysiak-Auvity, Thierry Le Diguarher, Jérôme-Benoît Starck, Ijen Chen, Neil Whitehead, Christopher Graham, Natalia Matassova, Pawel Dokurno, Christopher Pedder, Youzhen Wang, Shumei Qiu, Anne-Marie Girard, Emilie Schneider, Fabienne Gravé, Aurélie Studeny, Ghislaine Guasconi, Francesca Rocchetti, Sophie Maïga, Jean-Michel Henlin, Frédéric Colland, Laurence Kraus-Berthier, Steven Le Gouill, Martin J.S. Dyer, Roderick Hubbard, Mike Wood, Martine Amiot, Gerald M Cohen, John A. Hickman, Erick Morris, James Murray, Olivier Geneste

**Affiliations:** ^1^ Institut de Recherches Servier Discovery Chemistry Unit, Croissy Sur Seine, France; ^2^ Vernalis (R&D) Ltd., Cambridge, UK; ^3^ Institut de Recherches Servier Oncology R&D Unit, Croissy Sur Seine, France; ^4^ Institute for Experimental Cancer Research in Pediatrics, Goethe-University Frankfurt, Frankfurt, Germany; ^5^ Institut de Recherches Internationales Servier, Oncology R&D Unit, Suresnes, France; ^6^ Novartis Institute of Biomedical Research, Oncology Drug Discovery, Cambridge, MA, USA; ^7^ CRCINA, INSERM, CNRS, Université de Nantes, CHU de Nantes, Nantes, France; ^8^ Ernest and Helen Scott Haematological Research Institute, University of Leicester, Leicester, UK; ^9^ Institute of Translational Medicine, University of Liverpool, Liverpool, UK

**Keywords:** BCL-2, inhibitor, BH3-mimetics, apoptosis, hematological malignancies

## Abstract

Escape from apoptosis is one of the major hallmarks of cancer cells. The B-cell Lymphoma 2 (BCL-2) gene family encodes pro-apoptotic and anti-apoptotic proteins that are key regulators of the apoptotic process. Overexpression of the pro-survival member BCL-2 is a well-established mechanism contributing to oncogenesis and chemoresistance in several cancers, including lymphoma and leukemia. Thus, BCL-2 has become an attractive target for therapeutic strategy in cancer, as demonstrated by the recent approval of ABT-199 (Venclexta™) in relapsed or refractory Chronic Lymphocytic Leukemia with 17p deletion. Here, we describe a novel orally bioavailable BCL-2 selective and potent inhibitor called S55746 (also known as BCL201). S55746 occupies the hydrophobic groove of BCL-2. Its selectivity profile demonstrates no significant binding to MCL-1, BFL-1 (BCL2A1/A1) and poor affinity for BCL-XL. Accordingly, S55746 has no cytotoxic activity on BCL-XL-dependent cells, such as platelets. In a panel of hematological cell lines, S55746 induces hallmarks of apoptosis including externalization of phosphatidylserine, caspase-3 activation and PARP cleavage. *Ex vivo*, S55746 induces apoptosis in the low nanomolar range in primary Chronic Lymphocytic Leukemia and Mantle Cell Lymphoma patient samples. Finally, S55746 administered by oral route daily in mice demonstrated robust anti-tumor efficacy in two hematological xenograft models with no weight lost and no change in behavior. Taken together, these data demonstrate that S55746 is a novel, well-tolerated BH3-mimetic targeting selectively and potently the BCL-2 protein.

## INTRODUCTION

Apoptosis is a tightly controlled and evolutionarily conserved process of programmed cell death that is crucial for tissue homeostasis [1]. Evasion of apoptosis is critical for tumor development as well as maintenance and represents an almost universal hallmark of cancer which, importantly, also underpins resistance to diverse anti-cancer treatments [2]. BCL-2 family proteins are crucial regulators of the mitochondrial apoptotic pathway and are characterized by the presence of up to four conserved BCL-2 Homology (BH) domains. BCL-2, the first identified member of this family was originally discovered as part of the t(14;18) chromosomal translocation which occurs in human B cell lymphomas [3, 4]. BCL-2 family proteins are divided into 3 functionally distinct groups: (i) the pro-survival proteins (BCL-2, BCL-XL, BCL-W, MCL-1 and BFL-1/BCL2A1/A1), (ii) the multi-BH domain cell death effectors (BAX, BAK, BOK) and (iii) the BH3-only apoptosis initiators (*e.g.* BIM, BAD, NOXA) [5]. Dysregulation of the BCL-2 family altering the balance between pro-survival and pro-death members provides a common mechanism by which cancer cells acquire a survival advantage [6]. Dynamic interactions between members of BCL-2 family subgroups, involving binding of the BH3 domain of the pro-apoptotic members to a groove at the surface of the pro-survival proteins, control commitment to apoptosis [7]. Following activation by cellular stress, BH3-only proteins initiate apoptosis by inhibiting the pro-survival BCL-2 proteins [8] and potentially by directly activating BAX and BAK [9]. The subsequent activation and oligomerization of the death effectors BAX and BAK result in mitochondrial outer membrane permeabilization and apoptosis.

The pro-survival BCL-2 members exert their function by sequestering pro-apoptotic members through binding to their BH3 domains. Inhibiting these intracellular protein-protein interactions is therefore an attractive strategy to target the aberrant survival of cancer cells caused by BCL-2 family dysregulation [10]. ABT-737, which was discovered using structure-based drug design, targets BCL-2, BCL-XL and BCL-W and was the first example of an inhibitor of the pro-survival BCL-2 members demonstrating druggability of such targets [11]. Encouraging clinical activity against lymphoid malignancies thought to be BCL-2 dependent were observed in early trials with Navitoclax (ABT-263), the orally bioavailable analog of ABT-737 [12, 13, 14]. However, as BCL-XL has a crucial survival function in circulating platelets [15], Navitoclax induced a rapid thrombocytopenia due to its inhibition of BCL-XL, which limited its clinical utility. It was therefore hypothesized that a BCL-2 selective inhibitor should exhibit limited thrombocytopenia while maintaining antitumor activity in BCL-2 dependent lymphoid malignancies. Subsequent rational drug discovery research efforts led to the development of Venclexta™ (ABT-199), the first orally active BCL-2 selective (BCL-XL-sparing) inhibitor [16] that has been recently approved in high risk patients with relapsed or refractory Chronic Lymphocytic Leukemia (CLL) [17]. Here we describe S55746 (also known as BCL201), a novel, orally active BCL-2 specific inhibitor that has a partially overlapping but distinct BCL-2 hydrophobic groove binding mode compared to ABT-199. S55746, currently in phase I clinical trials in heamatological malignancies (Trials registration ID: NCT02920697, NCT02920541 and NCT02603445) displays all the hallmarks of a BCL-2 specific BH3-mimetic and exhibits robust antitumor activity in BCL-2 dependent lymphoid tumor xenograft models while sparing platelets.

## RESULTS

### S55746 is a selective inhibitor of BCL-2

**Figure 1:**
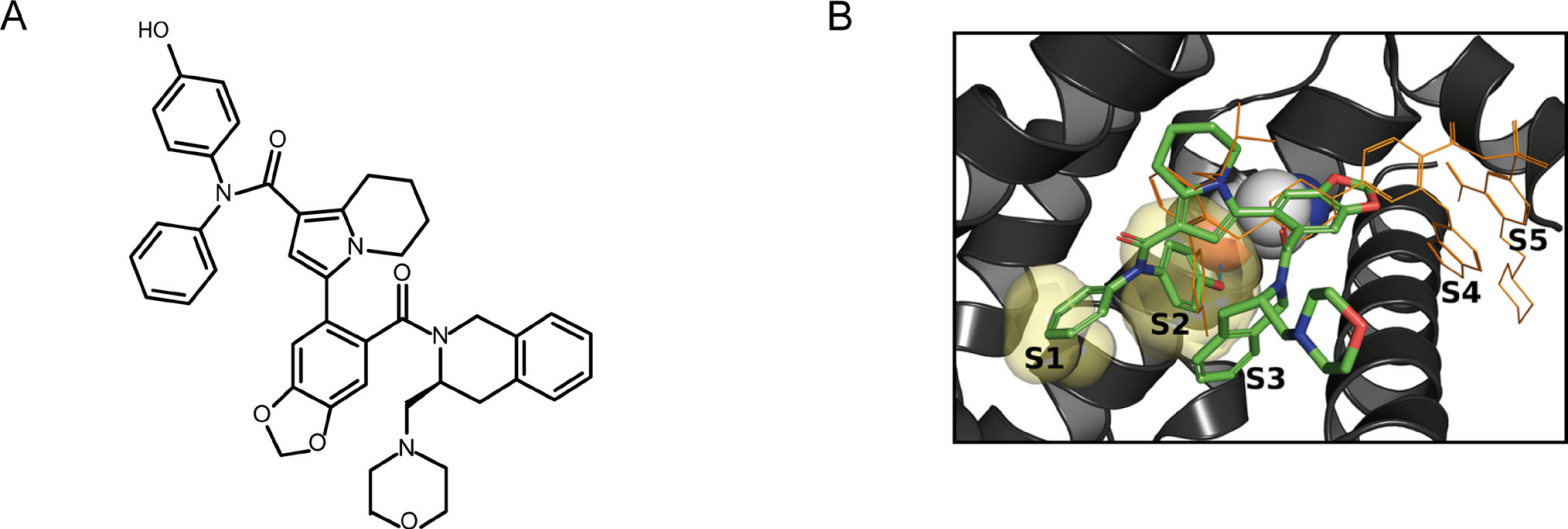
Chemical structure and binding mode of S55746 (**A**) Chemical structure of S55746. (**B**) Superposition of S55746 (green sticks) and ABT-199 analogue from PDB 4MAN (orange lines) X-ray crystal structures. Ala149 is highlighted (space-filling spheres) and the hydrogen bond from S55746 phenol to backbone carbonyl of Ala149 is shown (cyan dashed line). The key S1 and S2 binding pockets are shown as translucent yellow surfaces.By comparison with the ABT-199 analogue it can be seen that S55746 forms a compact structure that only accesses pockets S1/S2/S3 and not S4/S5. Image was prepared using Pymol.

Starting from a moderately active literature compound [18, 19] and using a structure-based drug design approach, S55746 was developped as described in Le Diguarher *et al.* 2013 [20] (Figure 1A and Supplementary Materials and Methods). Fluorescence polarization (FP) data, using Fluorescent-PUMA as a binder, demonstrates that S55746 is a potent inhibitor of BCL-2 (K_i_ = 1.3 nM). The selectivity of S55746 for BCL-2 versus BCL-XL ranges from ~70 to 400 folds depending on the assay used (Table 1). No significant binding to MCL-1 and BFL-1 was observed (Table 1). S55746 occupies the region typically referred to as S1/2/3 in contrast to the ABT-199 analog [16], which occupies a greater portion of the protein surface area including S2/3/4/5. S55746 adopts essentially the same binding mode as described in Porter *et al*. 2009 [18]. S55746 forms a single hydrogen bond to the backbone carbonyl of residue A149 (Figure 1B) buried deep into S2. The size-independent enthalpic efficiency (0.83) for S55746 binding to BCL-2 is suggestive of optimal polar and Van der Waal's interactions, indicative of highly specific binding (Figure 1B) [21].

**Table 1:** Associated affinity and selectivity data of ABT-263, S55746 and ABT-199

	FP_BCL-2_KI (M)	FP_BCL-XL_KI (M)	FP_MCL-1 (M)	FP_BFL-1 (M)	ITC_BCL-2_KD (M)	∆H (kcal/mol)	*N* (sites)	ITC_BCL-XL_KD (M)	SPR AIS_BCL-2_ KD (M)
**ABT-263**	4.5E-09	3.5E-09	9.4%@10E-06	NT	3.7E-09	–13.1	0.8	2.9E-09	2.1E-09
**S 55746**	1.3E-09	5.2E-07	4.3%@30E-06 4.5%@30E-06	7.4%@5E-06	2.47E-09	–13.5	1.1	1.86E-07	3.9E-09
**ABT-199**	1.2E-09	4.9E-08	14.9%@30E-06 11.8%@30E-06	6.4%@3E-05	ND	–12.1	1.1	2.1E-08	2.6E-10

The **KI** were determined by Fluorescence polarisation and **KD** by direct binding SPR. X%@10E-05 means X% of inhibition at the maximal tested concentration (M). FP: Fluorescence Polarization; SPR AIS: Surface Plasmon Resonance Affinity In Solution; ITC: Isothermal Titration Calorimetry; ∆H : enthalpy delta. NT, not tested. ND, not determinable (too potent). Of note, bottom limit for the FP assay was 1.2 nM.

### S55746 induces cell death selectively

**Figure 2:**
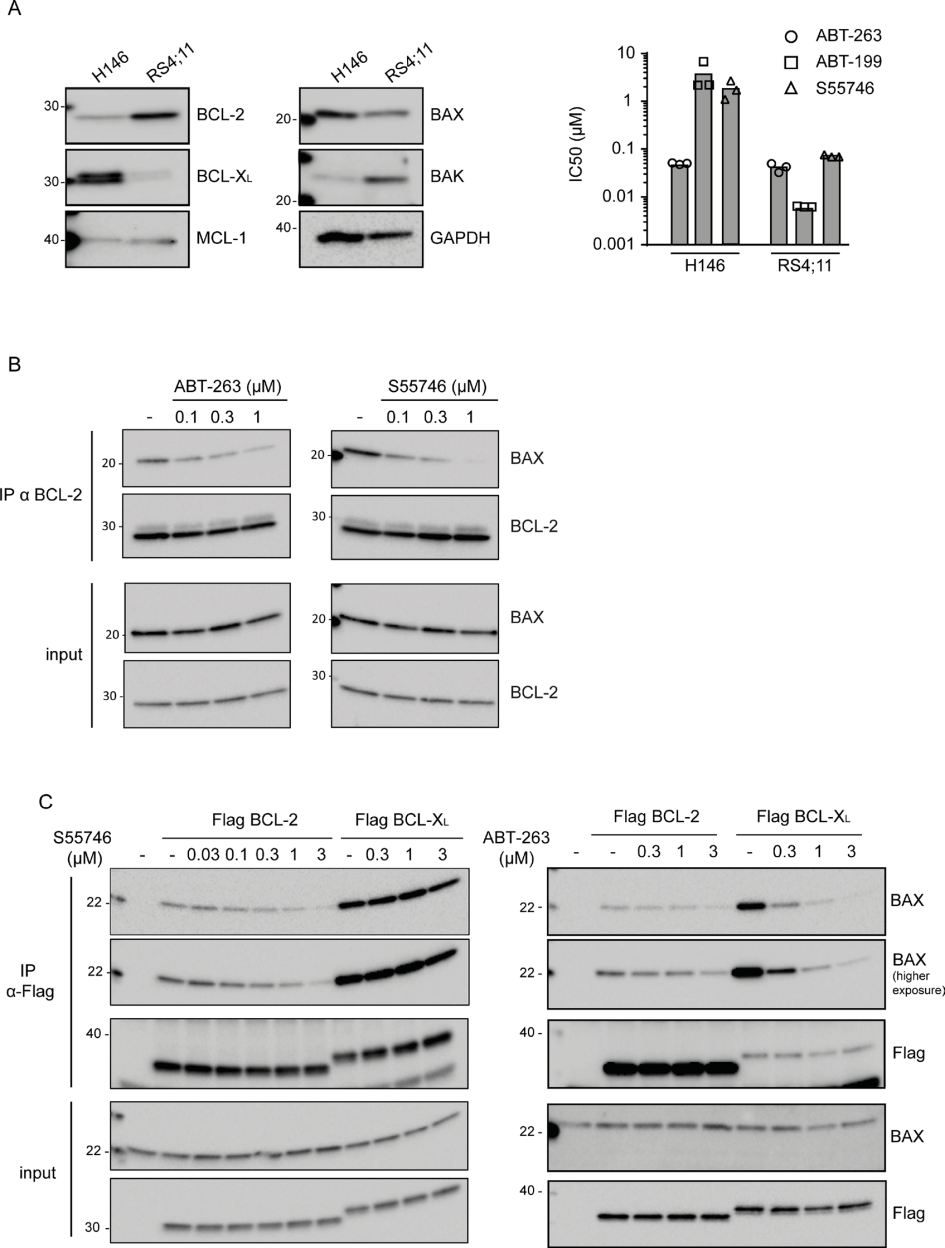
S55746 is a potent and selective inhibitor of BCL-2 (**A**) Expression of BCL-2, BCL-XL, MCL-1, BAX and BAK was assessed by immunoblotting in H146 and RS4;11 cell lines. GAPDH was used as a loading control (left panel). Viability of H146 and RS4;11 cell lines after incubation with increasing concentration of either ABT-263 (open circles), S55746 (open triangles) or ABT-199 (open squares) for 72 h. IC_50_ indicates concentration at which 50% of inhibition is reached. Mean and individual data of 3 independent experiments are shown (right panel). (**B**) Co-immunoprecipitation assay of RS4;11 cells treated with different concentrations of S55746 or ABT-263 for 2 h. ‘−’ indicates that cells were treated with DMSO only. Cells were lysed after treatment and immunoprecipitated (IP) with anti-BCL-2 antibody. The cell lysates (input) and immunoprecipitates were analyzed by immunoblotting with BAX or BCL-2 antibodies as indicated. (**C**) Co-immunoprecipitation assay of HeLa cells transfected with plasmids expressing Flag-tagged BCL-2 or Flag-tagged BCL-XL as indicated. Cells were treated with different concentrations of S55746 or ABT-263 for 2 h. ‘−’ indicates that cells were treated with DMSO only. Cells were lysed after treatment and immunoprecipitated (IP) with anti-Flag antibody. The cell lysates (input) and immunoprecipitates were analyzed by immunoblotting with BAX or Flag antibodies as indicated. Representative blots of three independent experiments are shown.

The selectivity and potency of S55746 was firstly evaluated in the well-described BCL-2-dependent acute lymphoblastic leukemia (ALL) RS4;11 cell line, which express high levels of BCL-2 but low levels of BCL-XL [22] (Figure 2A, left panel). S55746 potently induces RS4;11 cell killing after 72 h of treatment with an IC_50_ of 71.6 nM (Figure 2A, right panel and Supplementary Figure 1A). Interestingly, S55746 exhibits a much weaker activity in H146 (IC_50_ 1.7 μM), a BCL-XL-dependent cell line [23], which expresses a low level of BCL-2 and high level of BCL-XL (Figure 2A, left panel) whereas ABT-263, which targets BCL-2 and BCL-XL [14], induces equivalent cell killing in both RS4;11 and H146 (41.5 nM and 49.7 nM, respectively; Figure 2A, right panel and Supplementary Figure 1B). Of note, the BCL-2 selective inhibitor ABT-199 displays identical selectivity profile than S55746 in these two cell lines. Selective targeting of BCL-2 by S55746 was confirmed by co-immunoprecipitation experiments, which showed a concentration-dependent disruption of the BCL-2/BAX complex in RS4;11 cells following treatment with increasing concentrations of S55746 (Figure 2B). In BCL-2-overexpressing HeLa cells, S55746 affects BCL-2/BAX complex without any significant effect on the BCL-XL/BAX complex in BCL-XL-overexpressing HeLa cells (Figure 2C, left panel). In contrast, ABT-263 was shown to disrupt both BCL-2/BAX and BCL-XL/BAX complexes (Figure 2C, right panel).

### S55746 selectively induces apoptosis through BCL-2 inhibition in a BAX/BAK-dependent manner

**Figure 3:**
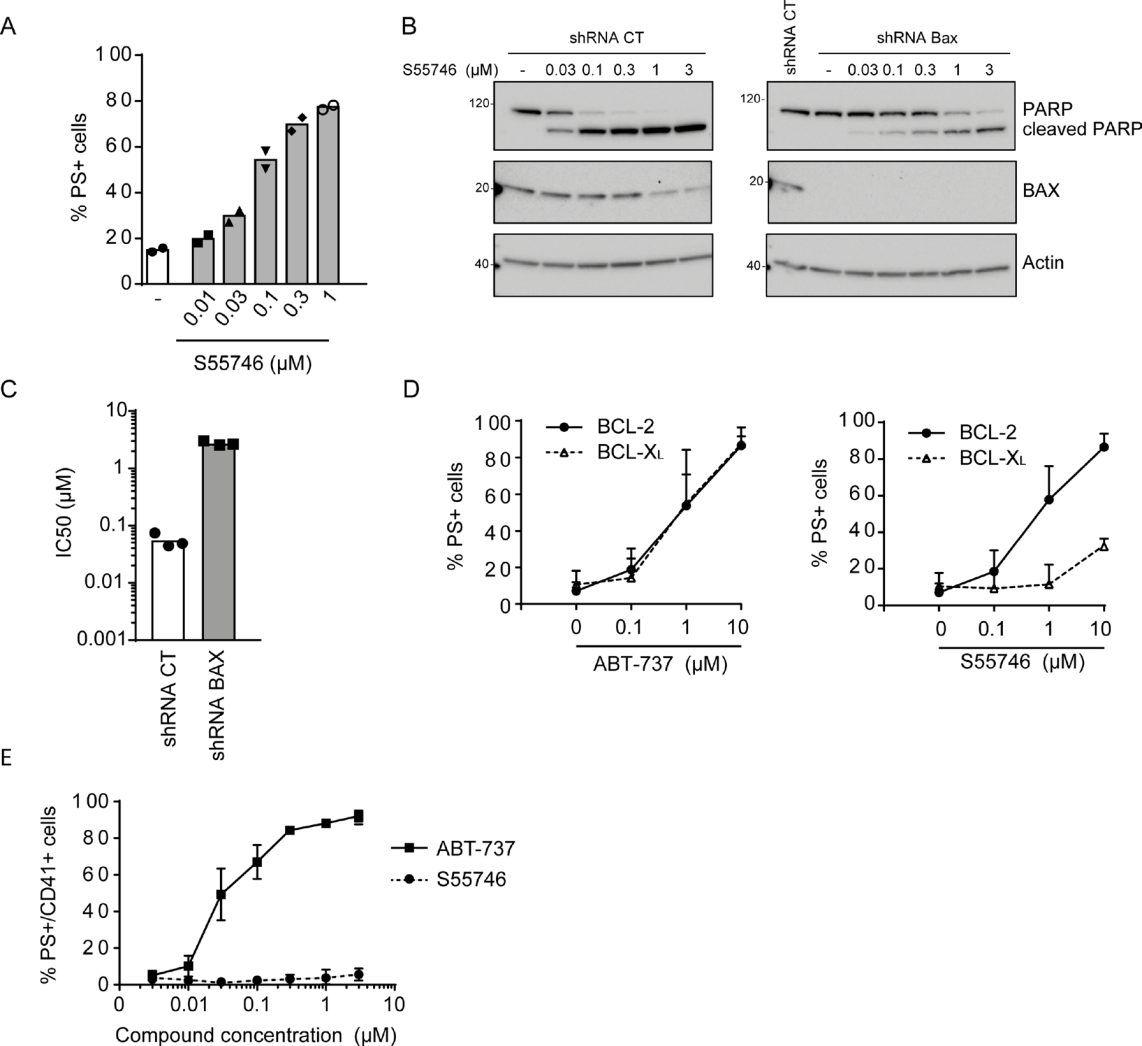
S55746 induces apoptosis in a BAX-dependent manner (**A**) Apoptosis induction in RS4;11 cells treated with S55746 at the indicated concentration for 2 h. Cells were analyzed by flow cytometry for PI and annexin V-FITC labeling. Mean and individual points from two biological replicates are shown. ‘−’ indicates that cells were treated with DMSO only. (**B**) Expression of PARP and BAX assessed by immunoblotting in RS4;11 cells transduced with shRNA CT (control) or shRNA BAX upon treatment with increasing concentrations of S55746. Actin was used as a loading control. ‘−’ indicates that cells were treated with DMSO only. (**C**) Viability of RS4;11 cells transduced either with shRNA CT or shRNA BAX treated with increasing concentration of S55746 for 72 h. IC_50_ indicates concentration at which 50% of inhibition is reached. Mean and individual points from *n* = 3 biological replicates are shown. (**D**) FL5.12 cells either expressing BCL-2 (filled circles, thick line) or BCL-XL (open triangles, dashed line) were treated with increasing concentration of either ABT-737 (left panel) or S55746 (right panel) for 24 h upon IL-3 withdrawal. Cells were then analyzed by flow cytometry for PI and annexin V-FITC labeling. Mean ± s.d. of 4 biological replicates are shown. (**E**) Isolated platelets from 4 healthy donors were treated with increasing concentrations of ABT-737 (squares, thick line) or S55746 (circles, dashed line) for 3 h. CD41-PE positive cells were then gated and analyzed for annexin V-FITC labeling. PS+: Phosphatidylserine positive cells. EC_50_: half maximal effective concentration.

S55746 rapidly induced apoptosis in a concentration dependent-manner, as monitored by externalization of phosphatidylserine (PS^+^ cells) in RS4;11 cells (Figure 3A). Cleavage of caspase-3 and Poly ADP-ribose polymerase (PARP) was also observed upon treatment by S55746 in a concentration dependent manner in the same cellular model (Supplementary Figure 1C and 1D, respectively). S55746-induced apoptosis in RS4;11 is mediated in part by the BAX effector protein since PARP cleavage (Figure 3B) is markedly inhibited in BAX-deficient RS4;11 cells, generated using a shRNA-based approach compared to control RS4;11 cells upon treatment with increasing concentration of S55746. The sensitivity of BAX-deficient RS4;11 cells was strongly reduced compared to control RS4;11 (≈ 50-fold, IC_50_ = 2.7 μM versus 0.057 μM, Figure 3C). The dependence of the killing activity of S55746 on BAX and BAK was further confirmed by CRISPR/Cas9 genome editing in the THP-1 AML cell line (Supplementary Figure 2).

To further assess the selectivity of S55746 on apoptosis induction, S55746 was tested in the IL3-dependent murine pro-B lymphocytic FL5.12 cell line expressing either BCL-2 or BCL-XL as a required protein for cell survival in the absence of IL-3 [24]. Upon IL-3 withdrawal, increasing concentrations of ABT-737, an analogue inhibitor of ABT-263 that targets BCL-2 and BCL-XL, efficiently induced apoptosis, as illustrated by the increasing percentage of PS^+^ cells in either BCL-2 or BCL-XL-dependent FL5.12 cells (Figure 3D, left panel). However, S55746 was shown to strongly induce apoptosis in BCL-2-dependent FL5.12 cells with a minor effect in BCL-XL-dependent FL5.12 cells (Figure 3D, right panel). Of note is that isolated platelets from healthy volunteers, known to be strictly dependent on BCL-XL for survival [15], were totally insensitive to S55746 (EC_50_ > 3 μM) in contrast to ABT-737 which strongly induced apoptosis (EC_50_ = 0.03 μM; Figure 3E). Altogether, these data demonstrate that S55746 kills cancer cells through on-target activity, meaning activation of a BAX/BAK-dependent mitochondrial apoptotic pathway by direct inhibition of the BCL-2 pro-survival protein.

### Activity of S55746 in hematological cell malignancies *in vitro* and *ex vivo*

**Figure 4:**
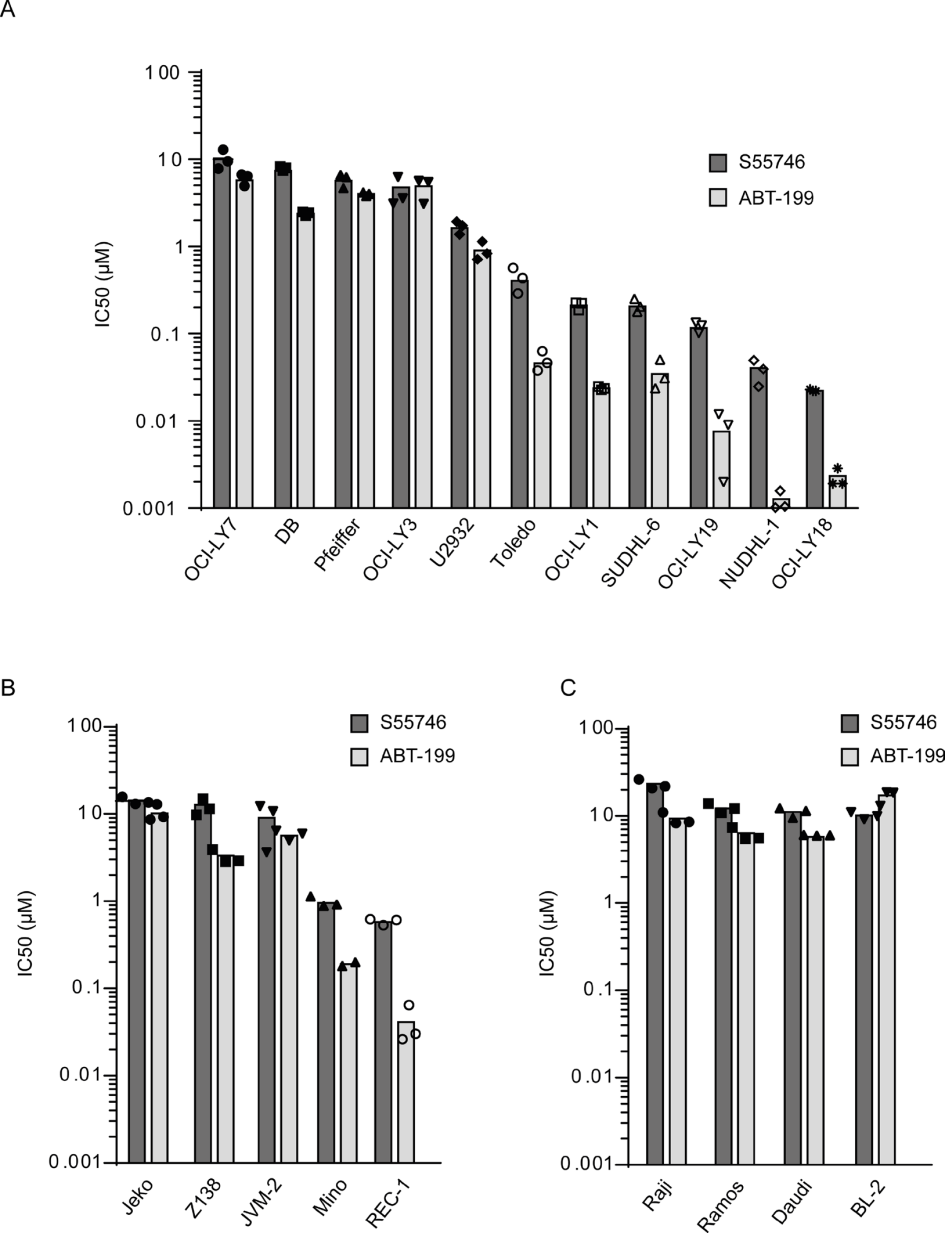
S55746 induces cell death in a panel of non-Hodgkin lymphoma cell lines Viability of a panel of Diffuse Large B-Cell (DLBCL, **A**), Mantle Cell Lymphoma (MCL, **B**) and Burkitt lymphoma (BL, **C**) cell lines treated with increasing concentration of S55746 and ABT-199 for 72 h was determined using Cell Titer Glo. IC_50_ indicates concentration at which 50% of inhibition is reached. Mean and individual points from *n* = 3 biological replicates are shown. Origins of the cell lines are described in supplementary materials and methods.

Activity of S55746 was assessed in a panel of non-Hodgkin lymphoma cell lines including Diffuse Large B-Cell Lymphoma (DLBCL; Figure 4A), Mantle Cell Lymphoma (MCL; Figure 4B) and Burkitt Lymphoma (BL; Figure 4C). After 72 h of treatment, we found that six out of eleven DLBCL cell lines tested had an IC_50_ below 1 μM. Two MCL cell line out of five tested displayed an IC_50_ below 1 μM for S55746. In contrast, all Burkitt lymphoma cell lines tested had an IC_50_ above 10 μM for S55746. Cellular results for the BCL-2 selective inhibitor ABT-199 are shown for comparison (Figure 4).

**Figure 5:**
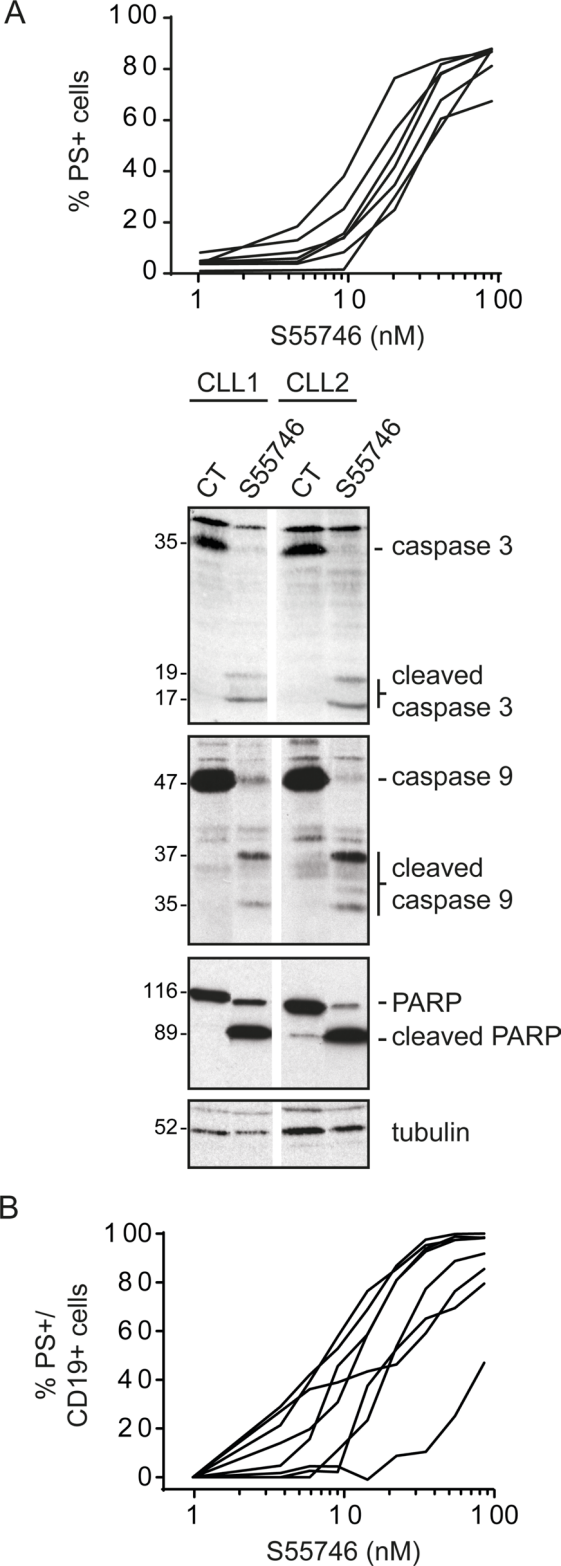
S55746 induces cell death in primary CLL and MCL cells (**A**) Primary Chronic Lymphocytic Leukemia (CLL) cells freshly isolated from 7 patients were treated for 4 h with increasing concentrations of S55746. Cells were then analyzed by flow cytometry for PI and annexin V-FITC labeling. Individual curves of cells treated with S55746 are shown (top panel). CLL cells isolated from 2 patients were exposed to S55746 (3 nM) for 4 h. Total and cleaved caspase-3, caspase-9 and PARP were detected by immunoblotting using specific antibodies. Tubulin was used as a loading control (lower panel). (**B**) Primary Mantle Cell Lymphoma (MCL) cells freshly isolated from 8 patients were cultured with increasing concentrations of S55746 for 24 h. Cells were then analyzed by flow cytometry for annexin V-FITC and CD19-APC positive labeling. Individual curves of cells treated with S55746 are demonstrated. PS+: Phosphatidylserine positive cells.

Chronic Lymphocytic Leukemia (CLL) cells are highly dependent on BCL-2 expression for survival [25]. Efficacy of S55746 was evaluated in primary CLL cells freshly isolated from 7 patients (Supplementary Table 1). S55746 caused rapid induction of apoptosis, reflected by the presence of phosphatidylserine positive cells. All primary CLL cells tested had an EC_50_ in a low nanomolar range (from 4.4 to 47.2 nM following 4 hours of treatment; Figure 5A, top panel and Supplementary Table 1). In two representative CLL patient samples, S55746 induced characteristic ultrastructural changes of apoptosis such as chromatin condensation (data not shown) in addition to extensive processing of caspase-9 and caspase-3 as well as cleavage of PARP (Figure 5A, lower panel). All these data are consistent with activation of the intrinsic apoptotic pathway in CLL cells by S55746. In contrast to the data obtained on cell lines (Figure 4B), S55746 potently induced apoptosis in primary MCL cells with EC_50_ ranging from 2.5 to 110 nM following 24h of S55746 treatment (Figure 5B and Supplementary Table 2). Of note, ABT-199 has also been shown to be more potent on primary MCL cells versus MCL cell lines ; this potency shift is likely due to higher expression of BCL- XL in cell lines compare to primary cells [26]. Altogether, these data demonstrate that S55746 is a potent BCL-2 inhibitor that induces apoptotic cell death in cells that are dependent on BCL-2 for survival.

### S55746 is an effective agent that induces tumor regression *in vivo*

**Figure 6:**
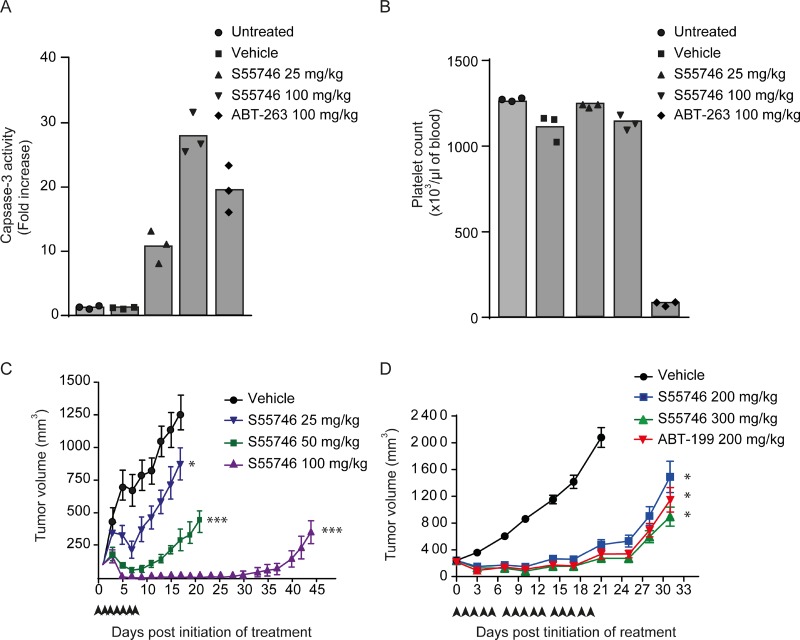
S55746 inhibits xenograft growth in RS4;11 and Toledo models (**A** and **B**) Female SCID mice were inoculated with 1 × 10^7^ RS4;11 cells and randomized 26 days after grafting. After randomization, mice were either not treated, or dosed once orally with vehicle, S55746 (25 mg/kg and 100 mg/kg) and ABT-263 (100 mg/kg). (**A**) Tumor samples were collected 16 h post dosing and analyzed for caspase-3 activity. Mean and individual data of caspase-3 activity fold increase over untreated group of 3 animals per treatment group are shown. (**B**) Platelet cells were counted 16 h post dosing. Mean and individual data of platelets counts of 3 animals per treatment group are shown. (**C**) Female SCID mice were inoculated with 1 × 10^6^ RS4;11 cells and randomized 27 days after grafting. After randomization, mice were either not treated (black circles) or treated orally with S55746 every day for 7 consecutive days at 25 mg/kg (blue reverse triangles), 50 mg/kg (green squares) or 100 mg/kg (purple triangles). Mean tumor volumes with error bars depicting s.e.m. of 8 animals per treatment group are shown. One way ANOVA on day 17, followed by Dunnett *post hoc* comparison was performed and always compared to vehicle, (^*^ *p* < 0.05; ^***^p < 0.001). (**D**) Female SCID mice were inoculated with 3 × 10^6^ Toledo cells and randomized 24 days after grafting. After randomization, mice were either not treated (black circles) or treated orally five times a week with ABT-199 at 200 mg/kg (inverted red triangles) and S55746 at 200 mg/kg (blue squares) or 300 mg/kg (green triangles) for 3 weeks. Mean tumor volumes with error bars depicting s.e.m. of 6–7 animals per treatment group are shown. One way ANOVA on day 21, followed by Dunnett *post hoc* comparison was performed and always compared to vehicle, (^*^ *p* < 0.05).

In order to confirm apoptosis induction by S55746 *in vivo*, caspase-3 activity was assessed on RS4;11 tumor xenografts 16 hours following single oral gavage treatment. Caspase-3 activity after S55746 treatment at 25 and 100 mg/kg was about 11 and 28 times higher than in vehicle-treated animals, respectively. ABT-263 induced caspase-3 activation about 20 times higher than in vehicle-treated group (Figure 6A). Consistent with the *in vitro* data (Figure 2 and Figure 3), S55746 treatment did not induce platelet loss *in vivo* at 25 and 100 mg/kg, while a strong decrease of platelet counts was observed upon treatment with ABT-263 at 100 mg/kg (Figure 6B).

Antitumor activity of S55746 was then evaluated in RS4;11 and Toledo models, two models that display different *in vitro* sensitivities towards S55746 (71.6 nM in RS4;11 (Figure 2A, right panel) *vs* 401 nM in Toledo (Figure 4A)). In RS4;11 bearing SCID mice, daily oral gavage treatment of S55746 for 7 consecutive days induced significant anti-tumor activity compared to untreated animals (*p* < 0.05 at 25 mg/kg and *p* < 0.001 at 50 and 100 mg/kg). This response increased in magnitude and in durability in a dose dependent manner (Figure 6C). Seventeen days after beginning of treatment at 25 mg/kg, 50 mg/kg and 100 mg/kg, tumor growth inhibition was 67.1, 16.3 and −93.8 T/C respectively, with complete regression observed in all animals treated at 100 mg/kg (Supplementary Figure 3A and Supplementary Table 3).

Efficacy of S55746 was also assessed in Toledo bearing mice treated by oral gavage five times a week for 3 weeks at 200 and 300 mg/kg and compared to ABT-199 at 200 mg/kg (Figure 6D). After 21 days of treatment, S55746 induced a significant tumor growth inhibition either at 200 or 300 mg/kg (13% and 2% T/C, respectively; *p* < 0.05; Supplementary Table 4). In this model, S55746 and ABT-199 showed similar anti-tumor efficacy (Figure 6D). Individual data at day 21 post-treatment are presented in Supplementary Figure 3B. Interestingly, body weight did not significantly differ in all treatment groups compared to the vehicle control group (Supplementary Figure 3C), even at doses up to 300 mg/kg of S55746. Altogether, these data indicate that S55746 is a highly efficacious and well-tolerated orally active BCL-2 inhibitor.

## DISCUSSION

In the present study, we describe S55746, a novel *bona fide* BH3 mimetic compound which selectively inhibits BCL-2. Importantly, S55746 induces the critical hallmarks of the mitochondrial apoptosis pathway and kills BCL-2 dependent tumor cells in a BAX/BAK dependent manner. *In vivo*, S55746 is highly efficacious against BCL-2 dependent tumor models without causing platelet loss, in agreement with its strong selectivity for BCL-2 over BCL-XL, thus supporting its evaluation in clinical trials.

The role of BCL-2 in lymphoid malignancies is well recognized and BCL-2 overexpression found in such diseases is driven by multiple mechanisms. The t(14;18) chromosome translocation, bringing the *BCL-2* gene under the control of the immunoglobulin heavy chain enhancer is found in all B-cell follicular lymphomas [3, 4] and in approximately 30% of DLBCL [27] while *BCL-2* gene amplification is commonly found in MCL and DLBCL [28]. BCL-2 is also overexpressed in both acute [29, 30] and chronic [31] leukemias where loss of microRNAs regulating BCL-2 expression are common [32, 33]. The clinical activity of Venclexta™ in these BCL-2 overexpressing hematological malignancies and its recent approval for patients with relapsed or refractory CLL with 17p deletion provide very strong clinical proof-of-concept and seminal registration for drugs targeting the BCL-2 family.

Target mutations causing resistance have been demonstrated with various high-affinity targeted therapies [34]. In the context of experimentally induced ABT-199 resistance, BCL-2 mutations affecting its hydrophobic groove have been identified [35, 36]. The clinical relevance of these mutations remains unknown at this stage. Since ABT-199 and S55746 have different binding modes, one can theoretically speculate that mutation(s) in the BCL-2 protein could cause resistance to one compound and not the other. Importantly, the pro-survival proteins MCL-1 and BCL-XL have also emerged as resistance factors to the BCL-2 specific inhibitor ABT-199 [37]. The recent discovery of selective BCL-XL [38, 39] and MCL-1 specific [40, 41] inhibitors is therefore of particular interest as these new specific BH3 mimetic compounds provide very promising strategies to overcome BCL-2 specific resistance mechanisms. The discovery of S55746, a new BCL-2 specific inhibitor, currently in phase I trials, could offer other opportunities to investigate the clinical utility of BCL-2 inhibition in the clinic.

## MATERIALS AND METHODS

### Human cells

Peripheral blood samples from CLL patients were obtained with informed patient consent and local ethical committee approval (UHL-09723). Peripheral blood mononuclear cells were purified using Histopaque (Sigma Aldrich, Dorset, United Kingdom) and cultured in RPMI 1640 medium supplemented with 10% FCS and 2 mM L-glutamine (Life Technologies Inc, Paisley, United Kingdom) at 2 × 10^6^ cells ml^-1^. Interphase fluorescence *in situ* hybridization (FISH) was performed for detection of 17p13 deletion. Mantle cell lymphoma (MCL) primary cells were obtained after informed consent from MCL patients treated at the department of clinical hematology from the University Hospital of Nantes, France. Peripheral MCL cells from blood or pleural effusion, were purified after Ficoll-Hypaque separation with immuno-magnetic anti-CD19 beads (Miltenyi, Paris, France). Primary cells were cultured in RPMI-1640 supplemented with 10% FCS and 2 mM glutamine. Human platelets were isolated from citrate blood donated by healthy volunteers. Washed platelets were cultured in Hepes-buffered saline (HBS, 10 mM Hepes, 150 mM NaCl, 5 mM KCl, 1 mM MgSO_4_, pH 7.4).

### Chemical

ABT-737, Venclexta™ (ABT-199) and ABT-263 were obtained from Selleck-Chem. Synthesis of S55746 is described in the supplemental material and methods.

### Fluorescence polarization (FP)

The assays were carried out in black-walled, flat bottomed, low binding, 384-well plates in buffer A (10 mM 4-(2-Hydroxyethyl)-1-piperazineethanesulfonic acid [HEPES], 150 mM NaCl, 0.05% Tween 20, pH 7.4 and 5% DMSO) in the presence of 10 nM Fluorescein-PUMA. Final concentrations of MCL-1, BFL-1, BCL-2 and BCL-XL proteins were 10, 10, 10 and 20 nM respectively. The assay plates were incubated for 2 hours at room temperature and the FP was measured on a Synergy 2 reader (Ex. 528 nm, Em. 640 nm, Cut-off 510 nm). The binding of increasing doses of compound was expressed as a percentage reduction in mP compared to the window established between ‘DMSO only’ and ‘total inhibition’ control (30 μM PUMA). The inhibitory concentrations that gave a 50% reduction in mP (IC_50_) were determined, from 11-point dose response curves, in XL-Fit using a 4-Parameter Logistic Model (Sigmoidal Dose-Response Model). The K_I_ was subsequently calculated as previously described [42].

### Surface plasmon resonance (AIS)

The experiments were performed at 30° C in a running buffer HBS-EP pH 7.4 (10 mM HEPES pH 7.4, 150 mM NaCl, 3 mM EDTA, 0.05% P20) supplemented 1 mM TCEP, 2% glycerol and 1% DMSO. PUMA BH3 peptide was purchased from Biopeptide LLC and immobilized on a CM-5 sensor chip. Standard amine coupling protocol of Biacore GE Healthcare has been applied with EDC/NHS coupling followed by ethanolamine deactivation. A channel treated in the same manner but in the absence of peptide was used as the reference channel. Compounds were tested in double-diluted series of twelve concentrations with top concentrations ranging from 20 nM to 2000 nM. Samples were preincubated with 1–2 nM BCL-2 in a running buffer and injected over the generated surfaces at a flow rate 15 μl/min for 300 sec. The calibration curve was generated using the same procedure but without added compound. Affinity evaluations were performed using the Affinity In Solution Model of BIAevaluation 2.1 (BIAcore GE Healthcare Bio-SciencesCorp) software.

### Isothermal titration calorimetry (ITC)

The protein was dialyzed prior to use in 2 L of 25 mM HEPES, 150 mM NaCl, 5% glycerol (v/v), 0.5% MTG (v/v) at pH 7.4 for 3 hours at room temperature. The protein was dialyzed using a 3.5 K MWCO Slide-A-Lyzer™ cassette. The protein solution was recovered, spin-filtered through a 0.22 μM frit. The filtered protein was quantified by UV absorbance at 280 nm using an extinction coefficient of 36440 M^-1^cm^-1^. 500 ml of dialysis buffer was retained (remainder used to rinse out SCHOT bottle) and degassed for a minimum of 30 mins under vacuum with constant stirring. Typically, 50 μM protein was titrated from the syringe into the cell containing 5 μM compound. The solutions were carefully matched to ensure same solvent and buffer conditions were present in both syringe and cell (this is to minimize heat of dilution effects). The final solution was the dialysis buffer plus 1% DMSO and 0.05% P20. The experiment was performed over 13 injections with stirring at 1000 rpm, gain setting low and at 25° C. Pure water was used in the reference cell. The first injection was 0.5 μl with a duration of 1 second with a gap of 150 seconds until the further 12 injections of 3.05 μl with a duration 6.1 seconds and a 240 second interval between injections. Data was analyzed using the vendor supplied software, PEAQ-ITC Analysis Software. The data model used was the single site model. The data shown is the average of 2 or 3 independent experiments. For compounds with affinities greater than 10 nM it is not possible to accurately determine the potency by ITC, however the enthalpy and stoichiometry are accurately determined. The data shows that each compound binds with a 1:1 stoichiometry and with high enthalpies on BCL-2 demonstrating a highly specific interaction.

### Crystallography

Crystals of the BCL-2 complex with S55746 were grown using BCL-2 (His6-BCL-2 based on the 2W3I construct with additional mutation G118Q) at a concentration of 9.4 mg/ml [20 mM Tris buffer pH 7.5, 100 mM NaCl, 2 mM DTT, 1% glycerol] by the hanging drop vapour diffusion technique. 2 μl of protein solution was mixed with 2 μl of crystallization reservoir (0.1 M sodium acetate buffer pH 5.25, 20% Jeffamine 600, 10% PEG3350) in a hanging drop plate. Crystallization was initiated by the addition of micro-crystals of the BCL-2/S55748 complex (S55748 is a methylated version of S55746). The new crystals, which grew over 2–3 days, were crushed and used for a second micro-seeding of a fresh mixture BCL-2/S55746 solution and crystallization reservoir. The plate was incubated at 284 K and bi-prism crystals appeared overnight and grew to a maximum ~50 μm along the edge over 4 days. Suitable crystals for data collection were flash frozen in liquid nitrogen after cryoprotection using crystallization reservoir. The diffraction data was collected at the Proximal station, Soleil Synchrotron, at wavelength 0.97857 Å using Pilatus-6 M detector. The crystal diffracted to 1.39 Å, P2_1_2_1_2_1_ space group and cell dimensions a = 35.09, b = 46.9, c = 85.04, α,β,γ 90,90,90 and mosaicity of 0.2 degrees and one molecule per asymmetric unit. Several cycles of refinement were conducted with Refmac5 software from the ccp4 package, alternating with manual rebuilding using the COOT software package. The final model consisted of 144 residues, S55746 ligand and 152 molecules of water. S55746 topology restraints were defined using the ProDrug software package.

### Cell sorting and flow cytometry analysis

CLL cells were exposed for 4 h with S55746 (1, 3, 10, 30, 100 nM) before analysis of apoptosis using staining with AnnexinV-FITC/propodium iodide and flow cytometry. EC_50_ values were calculated using GraphPadPrism (non-linear fit, sigmoidal dose response, variable slope). Washed human platelets were cultured at a final density of 2.5 × 10^7^/ml and exposed for 3 h with S55746 (3, 10, 30, 100, 300, 1000, 3000 nM) before staining with CD41-PE (BD) and Annexin-FITC and flow cytometry. Mononuclear MCL cells were cultured for 24 h in presence or absence of S55746 (concentration ranging from 0.1 nM to 100 nM) in RPMI supplemented with 10% FCS and 2 mM glutamine. Cell death was assessed in the CD19+ primary MCL cells by annexinV-FITC staining combined with an analysis of altered cellular morphology (lower FCS). Fluorescence was analyzed on FACSCalibur. The EC_50_ value was determined by the S55746 concentrations resulting in 50% of annexinV positive cells.

RS4;11 cells were treated with the indicated compounds for 2h, centrifuged and washed with binding buffer (10 mM Hepes, 140 mM NaCl, 2.5 mM CaCl_2_). Cells were incubated with 200 μl of binding buffer containing AnnexinV–FITC (Invitrogen) and propidium iodide (PI, Sigma) during 15 min at 20° C in the dark. THP-1 cells were incubated with 200 μl of binding buffer containing AnnexinV–APC (BD Biosciences) and DAPI (Sigma). 400 μl of binding buffer was added and samples were kept at 4° C before cytometric analysis. For each sample, 10^4^ cells were analyzed by flow cytometry on Epics XL/MCL flow cytometer (Beckman Coulter, France). Fluorescence was collected at 520 (FITC), 630 nm (PI), 660 nm (APC) and 470 (DAPI). Numbers of apoptotic cells (addition of primary apoptosis, secondary apoptosis and necrosis) was normalized to the total numbers of cells per tubes.

### CellTiter-Glo luminescent cell viability assay

Cells were seeded into 96-well plates and treated at 8 points with 1:2 serial dilution of compounds. Cell viability was assessed and assayed for viability using CellTiter-Glo reagent (Promega) following the manufacturer's instructions. Plates were read using Tecan luminescence plate reader. Results were normalized to the viability of cells without compounds (control wells). The IC_50_ values were calculated using nonlinear regression algorithms in XCell software.

### *In vivo* xenografts

Experiments were performed in SCID/beige female mice from Charles River Laboratories, Massachusetts, USA. Animals, approximately 7-8 weeks of age at the start of the treatment were allowed to acclimate in animal facility with access to food and water *ad libitum* for 3 days prior to manipulation. Animals were handled in accordance with IACUC regulations and guidelines for experiments performed in the USA and with European and French regulation for the protection of vertebrate animals for experiments performed in France. Animal well-being and behavior, including grooming and ambulation were monitored at least once a day. General health of mice was monitored and mortality recorded daily. Any moribund animals were sacrificed.

RS4;11 Acute Lymphoblastic Leukemia cell lines was obtained from ATCC. Toledo (Diffuse Large B-Cell Lymphoma) human cell lines were obtained internally through Bioresources and the Novartis-Cancer Cell Line Encyclopedia [43]. Cells were free of *Mycoplasma* and murine viral contamination in the IMPACT VIII PCR assay panel (IDEXX RADIL, IDEXX laboratories INC, Westbrook, ME, USA). Cells used for subcutaneous implantation were cultured in RPMI plus 10% FBS at 37° C in a humidified atmosphere containing 5% carbon dioxide). For RS4;11 culture, the medium was also supplemented with 2 mM L-glutamine, 100 U/ml penicillin, 100 μg/ml streptomycin, 10 mM Hepes and 4.5 g/L glucose. Cells were resuspended in a 1:1 mixture of cold DPBS (Dulbecco's Phosphate-Buffered Saline) and Matrigel^™^ (Becton-Dickinson #354234) at a concentration of 3 × 10^7^ cells/ml for Toledo cells, and of 1 × 10^7^ cells/ml for RS4;11 cells.

For each experiment, female SCID/beige mice were implanted subcutaneously (right axillary region) with 3 × 10^6^ Toledo or RS4;11 (1 × 10^6^ cells for efficacy studies and 1 × 10^7^ cells for pharmacodynamics studies) suspended in 1:1 mixture of cold DPBS and Matrigel^™^ in a total volume of 100 μl. Body weights were recorded and tumors were measured with digital calipers twice to three times a week. Tumor volume was calculated using the formula: length × width^2^/2. Percent changes in body weights was calculated as (BWcurrent - BWinitial)/(BWinitial) × 100. Data is presented as percent body weight change from the day of treatment initiation.

When tumors reached approximately 200 mm^3^ for efficacy studies or 300 mm^3^ for pharmacodynamic studies, mice were randomized. S55746 was formulated in PEG300/EtOH/water (40/10/50). ABT-199 was formulated in PEG300/EtOH/Phosal (30/10/60). Mice were treated via oral gavage at 10 ml/kg with the doses and schedules described in the figure.

For pharmacodynamic studies, blood and tumors were removed 16 h post dosing and immediately snap-frozen. Total proteins were extracted from the tumors and caspase-3 activity was assessed in triplicate using CaspACE^®^ Assay System (Promega). Platelet cell counts were determined 16 hours post dosing using Coulter AcT diff (Beckman).

For efficacy studies, percent treatment/control (T/C) values were calculated using the following formula: %T/C = 100 × ΔT/ΔC if ΔT > 0. Regression = 100 × ΔT/T_initial_ if ΔT < 0 where: T = mean tumor burden of the drug-treated group on the final day of the study; ΔT = mean tumor burden of the drug-treated group on the final day of the study – mean tumor burden of the drug-treated group on initial day of dosing; T_initial_ = mean tumor burden of the drug-treated group on initial day of dosing; C = mean tumor burden of the control group on the final day of the study; and ΔC = mean tumor burden of the control group on the final day of the study – mean tumor burden of the control group on initial day of dosing. If the tumor volume was less than 14 mm^3^ for more than 3 consecutive measurements, animals were considered in complete regression (CR).

### Statistical analysis

All *in vivo* data were expressed as mean ± standard error of the mean (SEM). Delta tumor volume and body weight were used for statistical analysis. Between-group comparisons were carried out using the Kruskal-Wallis ANOVA followed by a *post hoc* Dunn's test or Tukey's test. For all statistical evaluations, the level of significance was set at *p* < 0.05. Significance compared to the vehicle control group is reported unless otherwise stated.

## SUPPLEMENTARY MATERIALS FIGURES AND TABLES


