# Supplementary material for: S55746 is a novel orally active BCL-2 selective and potent inhibitor that impairs hematological tumor growth
Source: Oncotarget. 2018 Apr 13;9(28):20075–88. doi: 10.18632/oncotarget.24744 (PMC5929447; doi:10.18632/oncotarget.24744)
Supplement: Supplementary file 1 [file oncotarget-09-20075-s001.pdf]

S55746 is a novel orally active BCL-2 selective and potent inhibitor that impairs hematological tumor growth

SUPPLEMENTARY MATERIALS

S55746 synthesis

S55746 was synthesized as follows: All reagents obtained from commercial sources were used without further purification. Anhydrous solvents were obtained from commercial sources and used without further drying. The reactions were monitored by LC-MS, using BEH C18 column, gradient $CH_3CN/H_2O/CH_3SO_3H$ 0.1%. Flash chromatography was performed on ISCO CombiFlash Rf with pre-packed silica-gel cartridges. ^1H NMR and proton-decoupled ^{13}C NMR measurements were performed on Bruker Avance II 500 MHz spectrometer and Bruker Avance III HD 400 MHz spectrometer, using DMSO-d6 or $CDCl_3$ as solvent. ^1H and ^{13}C NMR data are in the form of delta values, given in parts per million (ppm) from tetramethylsilane (TMS), using the residual peak of the solvent as internal standard (DMSO-d6: 2.50 ppm (1 H)/39.5 ppm (13C); $CDCl_3$: 7.26 ppm (1 H)/77.0 ppm (13C)). Splitting patterns are designated as: s (singlet), d (doublet), t (triplet), m (multiplet), br (broad singlet), dd (doublet of doublets). Due to the high level of conformational restriction of such structures (and specifically from StepA), chemical shifts are given as mentioned before when possible. Otherwise, they are expressed as ranges. HRMS were determined on a Waters QTOF2 or ORBITRAP VELOS PROTHERMO.

Synthesis of intermediate 1

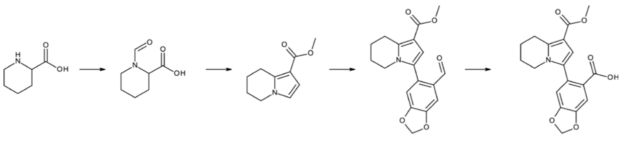

intermediate 1

Step 1.1: 1-formyl-2-piperidinecarboxylic acid

A solution of racemic 2-piperidinecarboxylic acid (40 g, 0.310 mol) in formic acid (300 mL) was chilled to 0° C, and then acetic anhydride (200 mL, 2.15 mol) was added dropwise. The reaction was stirred at room temperature overnight and then cooled to 0° C, quenched by water (250 mL) and stirred for 30 minutes at 0° C before being concentrated to dryness. The oil obtained was taken up in methanol (200 mL) and then concentrated to dryness again, giving 1-Formyl-2-piperidinecarboxylic acid as colorless oil (47.6 g, 0.303 mol, 98%).

^{1}H-NMR (400 MHz; DMSO-d6; 300K) δ: 13.0 (br, 1H); 8.0–8.05 (2s, 1H); 4.9–4.5 (2d, 1H); 4.1–2.6 (m, 2H); 2.2–1.2 (m, 6H).

Step 1.2: Methyl 5,6,7,8-tetrahydro-1-indolizinecarboxylate

To a solution of 1-formyl-2-piperidinecarboxylic acid (10 g, 63.6 mmol) in dichloroethane (65 mL) was added tosyl chloride (13.4 g, 70.4 mmol), methyl 2-chloroacrylate (11.5 mL, 113.5 mmol) and then dropwise, *N,N,N*-triethylamine (17.8 mL, 127.2 mmol). The reaction mixture was heated to reflux for 1.5 hours, cooled down to room temperature and methyl 2-chloroacrylate (5 mL, 48.9 mmol) and *N,N,N*-triethylamine (9 mL, 64 mmol) were added. The resultant mixture was heated to reflux overnight, diluted with methylene chloride, washed with a 1N HCl solution, saturated $NaHCO_3$ solution and brine. The organic phase was dried over $MgSO_4$, filtered, and concentrated to dryness. The residue was purified by flash chromatography (heptane/EtOAc gradient) to give Methyl 5,6,7,8-tetrahydro-1-indolizinecarboxylate (6.4 g, 56%) as a colorless oil.

^{1}H-NMR (400 MHz; $CDCl_3$; 300K) δ: 6.53 (d, 1 H), 6.41 (d, 1 H), 3.92 (t, 2 H), 3.78 (s, 3 H), 3.07 (t, 2 H), 1.95/1.85 (m, 4 H)

IR: νC = O 1692 cm^{-1} ester

Step 1.3: Methyl 3-(6-formyl-1,3-benzodioxol-5-yl)-5,6,7,8-tetrahydro-1-indolizine-carboxylate

To a solution of methyl 5,6,7,8-tetrahydro-1-indolizinecarboxylate (6.4 g, 35.7 mmol) in N,N-dimethylacetamide (12 mL) was added 6-bromo-1,3-benzodioxole-5-carbaldehyde (12.3 g, 53.6 mmol) and potassium acetate (7 g, 71.4 mmol). The reaction mixture was degassed over 20 min by bubbling a stream of argon. Bis(triphenylphosphine)palladium(II) dichloride (1.3 g, 1.8 mmol) was added and the reaction mixture was heated at 130° C for one hour and water (139 µL) was added. Heating was maintained at that same temperature overnight. The resultant mixture was diluted with AcOEt, activated charcoal (12 g) was added and the mixture was

stirred for one hour and filtered. The resulting organic phase was washed with water, dried over $MgSO_4$ and concentrated to dryness. The residue was purified by flash chromatography (heptane/EtOAc gradient) to give methyl 3-(6-formyl-1,3-benzodioxol-5-yl)-5,6,7,8-tetrahydro-1-indolizinecarboxylate (7 g, 70%) as a colorless oil.

^{1}H-NMR (400 MHz; DMSO-d6; 353K) d: 9.65 (s, 1H); 7.3 (s, 1H); 7.15 (s, 1H); 6.45 (s, 1H); 6.20 (s, 2H); 3.70 (s, 3H); 3.5-4.0 (m, 2H); 3.05 (m, 2H); 1.85 (m, 4H)

IR: νC = O 1695 cm^{-1} ester; νC = O 1674 cm^{-1}

Step 1.4: 6-[1-(Methoxycarbonyl)-5,6,7,8-tetrahydro-3-indolizinyl]-1,3-benzodioxole-5-carboxylic acid

To a 0° C solution of methyl 3-(6-formyl-1,3-benzodioxol-5-yl)-5,6,7,8-tetrahydro-1-indolizinecarboxylate (3.37 g, 10.3 mmol) in acetone (9.3 mL) and 2-methyl-2-butene (8.8 mL, 80.24 mmol) was added dropwise an aqueous solution of sodium chlorite (3.3 g, 36.05 mmol), sodium phosphate monobasic (NaH_2PO_4, 3.6 g, 25.75 mmol) in water (9.3 mL). The reaction mixture was stirred at room temperature for 7 hours, and concentrated under reduced pressure to remove acetone. The precipitate was filtered, washed with water and then dried (*in vacuo* at 40° C overnight) to give 6-[1-(methoxycarbonyl)-5,6,7,8-tetrahydro-3-indolizinyl]-1,3-benzodioxole-5-carboxylic acid (3.50 g, quantitative) as a white powder

^{1}H-NMR (400 MHz; DMSO-d6; 300K) d: 12.10 (m, 1H); 7.40 (s, 1H); 6.88 (s, 1H); 6.20 (s, 1H); 6.18 (s, 2H); 3.70 (s, 3H); 3.55 (t, 2H); 3.00 (t, 2H); 1.80 (m, 4H)

IR: νOH: 3000–2000 cm^{-1}acid; νC = O 1686–1676 cm^{-1}ester+acid; νC = C1608 cm^{-1}

Synthesis of intermediate 2

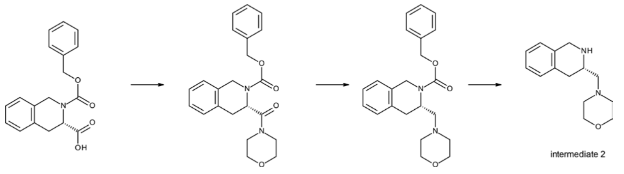

Step 2.1: Benzyl (3S)-3-(4-morpholinylcarbonyl)-3,4-dihydro-2(1H)-isoquinoline carboxylate

To a solution of (3S)-2-[(benzyloxy)carbonyl]-1,2,3,4-tetrahydro-3-isoquinolinecarboxylic acid (5 g, 16 mmol) in dichloromethane (160 mL) was added morpholine (1.5 mL, 17.6 mmol), N,N,N-triethylamine (9 mL, 64 mmol), N-(3-Dimethylaminopropyl)-N′-ethylcarbodiimide hydrochloride (3.3 g, 19.2 mmol) and hydroxybenzotriazole (2.6 g, 19.2 mmol). The reaction mixture was stirred at room temperature overnight, poured into an saturated aqueous solution of ammonium chloride and extracted with ethyl acetate. The combined organic layers were dried over $MgSO_4$, filtered and evaporated to dryness. The residue was purified by flash chromatography (dichloromethane/methanol gradient) to give benzyl (3S)-3-(4-morpholinylcarbonyl)-3,4-dihydro-2(1H)-isoquinoline carboxylate (5.3 g, 87%) as a white solid.

^{1}H-NMR (400 MHz; DMSO-d6; 353K) δ: 7.30 (m, 5H); 7.15 (m, 4H); 5.2–5.0 (m, 3H); 4.75–4.5 (2d, 2H); 3.55–3.3 (m, 8H); 3.15–2.9 (2dd, 2H)

IR: νC = O: 1694; 1650 cm^{-1}

Step 2.2: Benzyl (3S)-3-(4-morpholinylmethyl)-3,4-dihydro-2(1H)-isoquinoline carboxylate

To a solution of benzyl (3S)-3-(4-morpholinylcarbonyl)-3,4-dihydro-2(1H)-isoquinoline carboxylate (5.3 g, 13.9 mmol) in THF (278 mL) was added a 2M solution of BH_3Me_2S (14 mL, 27.8 mmol) in THF at room temperature. The reaction mixture was heated for 4 hours at 80° C, cooled down to room temperature, BH_3Me_2S 2 M in THF was added again (7 mL, 14 mmol). The reaction mixture was again heated at 80° C for 2 hours and concentrated under reduced pressure and slowly diluted with methanol (20 mL) and 5N hydrochloric acid (5.6 mL, 27.8 mmol). The mixture was stirred at room temperature overnight and for one hour at 80° C. The resultant mixture was neutralized carefully adding saturated $NaHCO_3$ solution at 0° C until pH 8 and extracted with AcOEt. The combined organic layers were dried over $MgSO_4$, filtrered and evaporated to dryness, giving crude benzyl (3S)-3-(4-morpholinylmethyl)-3,4-dihydro-2(1H)-isoquinoline carboxylate (4.9 g, 96%) as a colorless oil that was used without further purification in the next step.

^{1}H-NMR: (400 MHz; DMSO-d6; 353K) δ: 7.43–7.30 (m, 5H); 7.19 (m, 4H); 5.16 (m, 2H); 4.79–4.29 (d, 2H); 4.58 (m, 1H); 3.50 (m, 4H); 3.02–2.80 (dd, 2H); 2.42–2.28 (m, 5H); 2.15 (dd, 1H)

IR: νCH: 2810 cm^{-1}; νC = O: 1694 cm^{-1}; νC-O-C<: 1114 cm^{-1}; νCH-Ar: 751; 697 cm^{-1}

Step 2.3: (3S)-3-(4-Morpholinylmethyl)-1,2,3,4-tetrahydroisoquinoline

To a solution of benzyl (3S)-3-(4-morpholinylmethyl)-3,4-dihydro-2(1H)-isoquinoline carboxylate (4.9 g, 13.4 mmol) in ethanol (67 mL) was added palladium hydroxide on charcoal (20% Pd w/w, 0.980 g). The reaction mixture was stirred under H_2 atmosphere (1.2 bar) at room temperature for 4 hours, filtered and the filtrate was evaporated to dryness, affording (3S)-3-(4-Morpholinylmethyl)-1,2,3,4-tetrahydroisoquinoline (2.95 g, 95%) as a colorless oil.

^{1}H-NMR (400 MHz; DMSO-d6; 300K) δ: 7.12–7.0 (m, 4H); 3.92 (s, 2H); 3.60 (t, 4H); 2.98 (m, 1H); 2.68 (dd, 1H); 2.5–2.3 (m, 8H).

IR: νNH: 3322 $cm^{-1;}$ νC-O-C<: 1115 $cm^{-1;}$ nCH-Ar: 742 cm^{-1}

HRMS (ESI+): calculated for $C_{14}H_{21}N2O$ 233.1648, found 233.1645 ($[M+H]^+$)

Synthesis of intermediate 3: 4-[(tert-butyldimethylsilyl)oxy]-N-phenylaniline

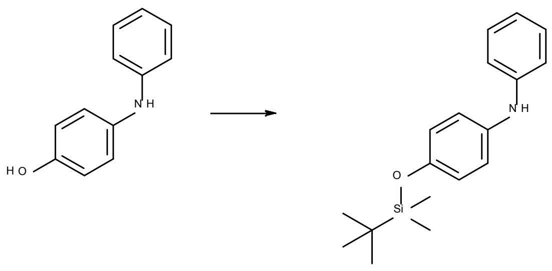

intermediate 3

To a solution of 4-anilinophenol (12 g; 64.7 mmol) in acetonitrile (200 mL) was added at room temperature imidazole (6.7 g, 97.05 mmol) and *tert*-butyl(chloro) dimethylsilane (11.7 g, 77.64 mmol). The reaction mixture was heated at 70° C for 4 hours, poured into water and extracted with ether. The organic phases were combined, dried over $MgSO_4$, filtered and concentrated *in vacuo*. Purification by flash column chromatography (petroleum ether/dichloromethane gradient) afforded 4-[(tert-butyl-dimethylsilyl)oxy]-N-phenylaniline (18.5 g, 95%) as a white powder.

^{1}H-NMR (400 MHz; DMSO-d6; 300K) δ: 7.84 (s, 1H); 7.17 (t, 2H); 6.98 (d, 2H); 6.94 (d, 2H); 6.76 (d, 2H); 6.72(t, 1H); 0.95 (s, 9H); 0.15 (s, 6H)

IR: νNH: 3403 cm^{-1}; νC = CAr: 1597 cm^{-1}

HRMS (ESI+): calculated for $C_{18}H_{25}NOSi$ 300.1778, found 300.1777 ($[M+H]^+$)

Final steps:

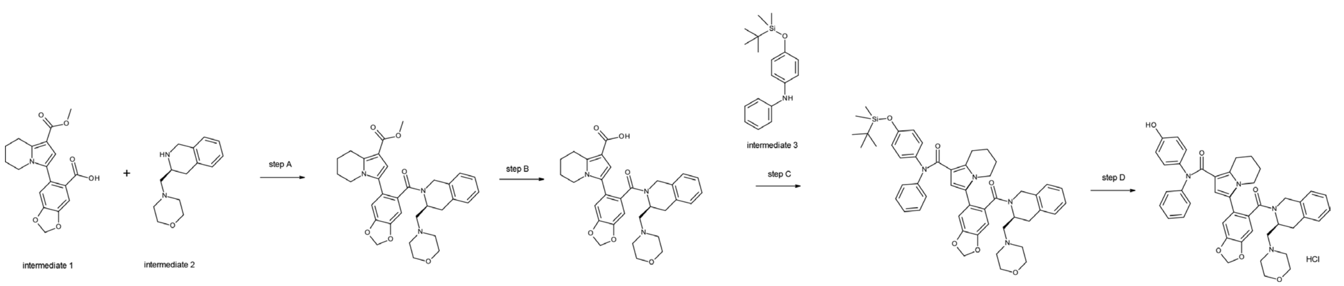

Step A: Methyl 3-{6-[((3S)-3-(4-Morpholinylmethyl)-3,4-dihydro-2(1H)-isoquinolinyl)carbonyl]-1,3-benzodioxol-5-yl}-5,6,7,8-tetrahydro-1-indolizinecarboxylate

To a solution of 6-[1-(methoxycarbonyl)-5,6,7,8-tetrahydro-3-indolizinyl]-1,3-benzodioxole-5-carboxylic acid (2 g, 5.83 mmol) in dichloromethane (20 mL) was added at room temperature N,N,N-triethylamine (5.5 mL, 6.96 mmol), (3S)-3-(4-morpholinylmethyl)-1,2,3,4-tetrahydroisoquinoline (2.12 g; 6.96 mmol), hydroxybenzotriazole (0.94 g, 6.96 mmol) and of N-(3-Dimethylaminopropyl)-N′-ethylcarbodiimide hydrochloride (1.4 g, 6.96 mmol). The reaction mixture was stirred at room temperature overnight, poured into an aqueous saturated ammonium chloride solution and extracted with ethyl acetate. The organic phase was dried over $MgSO_4$, filtered and concentrated *in vacuo*. The residue was purified by flash chromatography (heptane/AcOEt gradient) to give methyl 3-{6-[((3S)-3-(4-Morpholinylmethyl)-3,4-dihydro-2(1H)-isoquinolinyl)carbon yl]-1,3-benzodioxol-5-yl}-5,6,7,8-tetrahydro-1-indolizinecar-boxylate (2 g, 61%) as a white solid.

^{1}H-NMR: (500 MHz; DMSO-d6; 300K) δ: 7.2-6.9 (m, 4H); 7.04-7.03-7.00 (m, 1H); 6.85 (m, 1H); 6.35-6.06 (m, 1H); 6.15-6.12 (m, 2H); 5.06-4.84 (m, 1H); 4.86-4.17 (m, 2H); 3.65-3.55 (m, 3H); 3.43-4.26 (m, 2H); 3.58-3.5 (m, 4H); 2.37-3.05 (m, 4H); 1.68-2.56 (m, 4H); 1.4-2.0 (m, 4H)

IR: νC = O 1695 cm^{-1}ester; νC = O 1625 cm^{-1} amide; νC-O-C 1214-1176-1115 cm^{-1}; γCH-Ar 772-744 cm^{-1}

Step B: Lithium 3-[6-[(3S)-3-(morpholinomethyl)-3,4-dihydro-1H-isoquinoline-2-carbonyl]-1,3-benzodioxol-5-yl]-5,6,7,8-tetrahydroindolizine-1-carboxylate

To a solution of methyl 3-{6-[((3S)-3-(4-mor-pholinylmethyl)-3,4-dihydro-2(1H)-isoquinolinyl)carbon yl]-1,3-benzodioxol-5-yl}-5,6,7,8-tetrahydro-1-indolizi-necarboxylate (4.6 g, 8.26 mmol) in dioxane (24 mL) was added lithium hydroxide monohydrate (675 mg, 16.1 mmol). The reaction mixture was heated by microwave irradiation for 3 h at 100° C (140 W), filtrered, evaporated to dryness and dried under vacuum in the presence of P_2O_5 at 40° C overnight, affording lithium 3-[6-[(3S)-3-(morpholinomethyl)-3,4-dihydro-1H-isoquin oline-2-carbonyl]-1,3-benzodioxol-5-yl]-5,6,7,8-tetrahy-droindolizine-1-carboxylate (4.5 g, quantitative yield), which was used in the next chemical step without purification.

^{1}H-NMR: δ (400 MHz; DMSO-d6; 353K): 6.7–7.15 (m, 6H); 6.21 (s, 1H); 6.03 (s, 2H); 4.0–5.0 (m, 3H); 3.4–3.6 (m, 6H); 2.5–3.1 (m, 4H); 1.5–2.4 (m, 10H).

IR: νC = O: 1567 cm^{-1} broad; νC-O-C 1236 cm^{-1}.

HRMS (ESI+): calculated for $C_{31}H_{33}LiN_3O_6$ 550.2518, found 550.2524 ($[M+H]^+$)

Step C: N-[4-[tert-Butyl(dimethyl)silyl]oxyphenyl]-3-[6-[(3S)-3-(morpholinomethyl)-3,4-dihydro-1H-isoquinoline-2-carbonyl]-1,3-benzodioxol-5-yl]-N-phenyl-5,6,7,8-tetrahydroindolizine-1-carboxamide

To a 0° C solution of 3-[6-(3-morpholin-4-yl methyl-3,4-dihydro-1H-isoquinoline-2-carbonyl)-benzo[1,3]dioxo-5-yl]-5,6,7,8-tetrahydro-indolizine-1-lithium carb oxylate (2.6 g, 4.73 mmol) in dichloromethane (47 mL) was added dropwise oxalyl chloride (1.2 mL, 14.1 mmol). After stirring at room temperature for 11 hours, the reaction mixture was evaporated under reduced pressure. The residue was dissolved in dichloromethane (10 mL) and evaporated to dryness. This was repeated two times and then the residue was diluted with dichloromethane (37 mL) and a solution of 4-[(tert-butyldimethylsilyl)oxy]-N-phenylaniline (2.1g, 7.1mmol) and pyridine (0.6 mL, 7.1 mmol) in dichloromethane (10 mL) was added. The reaction mixture was stirred at room temperature overnight, concentrated under reduced pressure and purified by flash column chromatography on silica (dichloromethane/methanol gradient) to afford N-[4-[tert-butyl(dimethyl)silyl]oxyphenyl]-3-[6-[(3S)-3-(morpholinomethyl)-3,4-dihydro-1H-isoquinoline-2-carbonyl]-1,3-benzodioxol-5-yl]-N-phenyl-5,6,7,8-tetrahydroindolizine-1-carboxamide (1.9 g, 49%) as a beige solid.

^{1}H-NMR: δ (400 MHz; DMSO-d6; 353K): 6.6–7.25 (m, 16H), 6.03 (s, 2H), 4.0–5.3 (m, 3H), 3.4–3.8 (m, 6H), 2.5–2.9 (m, 4H), 1.95–2.45 (m, 6H), 1.60–1.70 (m, 4H), 0.9 (s, 9H), 0.1 (s, 6H).

IR: νC = O: 1632 cm^{-1}; νC-O-C<: 1237 cm^{-1}; νSi-O-C-: 910 cm^{-1}; νSi-C: 838/825 cm^{-1}; γCH-Ar: 806 cm^{-1}

HRMS (ESI+): calculated for $C_{49}H_{57}N_4O_6Si$ 825.4042, found 825.4052 ($[M+H]^+$)

Step D: N-(4-Hydroxyphenyl)-3-[6-[(3S)-3-(morpholinomethyl)-3,4-dihydro-1H-isoquinoline-2-carbonyl]-1,3-benzodioxol-5-yl]-N-phenyl-5,6,7,8-tetrahydroindolizine-1-carboxamide hydrochloride

To a solution of N-[4-[tert-butyl(dimethyl)silyl]oxyphenyl]-3-[6-[(3S)-3-(morpholinomethyl)-3,4-dihydro-1H-isoquinoline-2-carbonyl]-1,3-benzodioxol-5-yl]-N-phenyl-5,6,7,8-tetrahydroindolizine-1-carboxamide (1.9 g, 2.3 mmol) in methanol (4 mL) was added a solution of potassium hydroxide (0.646 g , 11.5 mmol) in methanol (8 mL). The reaction mixture was stirred for 30 minutes at room temperature, diluted with dichloromethane, washed successively with 1N HCl solution, saturated $NaHCO_3$ solution and brine. The organic phase was dried over $MgSO_4$, filtered and concentrated *in vacuo*. The residue was purified by flash chromatography on silica (dichloromethane/methanol gradient) to give N-(4-hydroxyphenyl)-3-[6-[(3S)-3-(morpholinomethyl)-3,4-dihydro-1H-isoquinoline-2-carbonyl]-1,3-benzodioxol-5-yl]-N-phenyl-5,6,7,8-tetrahydroindolizine-1-carboxamide (1.3 g, 1.9 mmol) as a white solid.

The corresponding hydrochloride salt was obtained by diluting the free base in dichloromethane and addition of a solution of 1 M HCl in ether (2 mL, 2 mmol), after 1h evaporation to dryness, solubilization in MeCN/H_2O and freeze-drying, affording N-(4-hydroxyphenyl)-3-[6-[(3S)-3-(morpholinomethyl)-3,4-dihydro-1H-isoquinoline-2-carbonyl]-1,3-benzodioxol-5-yl]-N-phenyl-5,6,7,8-tetrahydroindolizine-1-carboxamide hydrochloride (1.37 g, 1.8 mmol) as a white solid.

^{1}H NMR: (500 MHz, DMSO-d6) δ ppm 11.1 (bs, 1H), 9.44 (bs, 1H), 7.43 (m, 1 H), 7.3-6.87 (m, 9 H), 6.83 (m, 1 H), 6.7 (m, 2 H), 6.61 (m, 2 H), 6.1 (m, 2 H), 5.26 (m, 1 H), 5 (m, 1 H), 4.69/4.19 (m, 2 H), 4/3.89 (m, 4 H), 3.74/3.4/3.12/3 (m, 4 H), 3.68/3.29 (m, 2 H), 3.21/3.13 (m, 2 H), 2.85/2.44 (m, 2 H), 2.69/2.62 (m, 2 H), 1.59 (m, 2 H), 1.5/1.41 (m, 2 H)

^{13}C NMR 13C (500 MHz, dmso-d6) δ ppm 130-123, 129, 115.6, 110.1, 110, 107.8, 101.6, 62.8, 55.4, 52.5/50.3, 44.6, 43.7, 41.3, 30.7, 24.1, 22.1, 19.4

IR: νOH : 3100 à 3550 cm^{-1} phenol+ H2O; nNH$^+$: 2200 à 2700 cm^{-1}; νC=O: 1624 cm^{-1} amide; ν C-O: 1239 cm^{-1}

Elemental analysis:

Calculated (%) for $C_{43}H_{42}N_4O_6$ HCl: C 69.11, H 5.8, N 7.5, Cl- 4.74

Measured: C 68.95, H 5.46, N 7.51, Cl- 4.48.

HRMS (ESI+): calculated for $C_{43}H_{42}N_4O_6$ 711.3183, found 711.3163 ([M+H]+)

Optical rotation: $(\alpha)_D^{20}$ = + 50.8° $cm^3\ g^{-1}\ dm^{-1}$ (c = 9 mg/ml in MeOH)

Chiral purity: Purity >99% (SFC, using Cellocoat 3µm 4.6 × 150 mm column, eluent: CO_2/(methanol/diethylamine: 100/0.5): 70/30), by comparison with the R enantiomer.

Cell line culture

All cell lines were grown at 37° C in a humidified atmosphere with 5% CO_2. RS4;11 (ATCC® CRL1873™), HeLa (ATCC® CCL-2™), Pfeiffer (ATCC® CRL-2632™), Toledo (ATCC® CRL-2631™), SUDHL-6 (ATCC® CRL 2959™), DB (ATCC® CRL-2289™), REC-1 (ATCC® CRL-3004™), JVM-2 (ATCC® CRL-3002™), H146 (ATCC® HTB-173™), Daudi (ATCC® CCL-213™), Raji (ATCC® CCL-86™), Ramos (ATCC® CRL-1596™), JeKo-1 (ATCC® CRL-3006™), Z-138 (ATCC® CRL-3001™) and THP-1 ATCC® TIB-202™) were purchased from American Type Culture Collection (ATCC). OCI-LY18, Mino, U2932, NUDHL-1, OCI-LY3, BL-2, OCI-LY19, OCI-LY7, OCI-LY1 were from Leibniz-Institute DSMZ (Braunschweig, Germany). RS4;11 and THP-1

cells were cultured in RPMI 1640 medium supplemented with 10% heat inactivated fetal bovine serum (FBS), 2 mM L-glutamine, 100 U/ml penicillin, 100 µg/ml streptomycin, and 10 mM Hepes, pH 7.4. The medium was supplemented with 4.5 g/L glucose. HeLa cells were cultured in DMEM medium (containing 10% heat inactivated FBS, 10 mM Hepes, 100 U/mL penicillin, 100 µg/ml streptomycin). Pfeiffer, OCI-LY18, Toledo, U2932, NUDHL-1, SUDHL-6, DB, REC-1, JVM-2, H146, Daudi, Raji and Ramos cells were cultured in RPMI 1640 medium supplemented with 10% heat inactivated FBS, 2 mM L-glutamine, 100 U/ml penicillin, 100 µg/ml streptomycin, and 10 mM Hepes, pH 7.4. OCI-LY3, BL-2 and Jeko cells were cultured in the same medium supplemented with 20% FBS. Mino were cultured in the same medium supplemented with 15% FBS. OCI-LY19 cells were cultured in Alpha MEM medium supplemented with 10% heat inactivated FBS, 2 mM L-glutamine, 100 U/ml penicillin, 100 µg/ml streptomycin, and 10 mM Hepes, pH 7.4. OCI-LY7 and Z-138 cells were cultured in IMDM medium supplemented with 10% heat inactivated FBS, 2 mM L-glutamine, 100 U/ml penicillin, 100 µg/ml streptomycin, and 10 mM Hepes, pH 7.4. OCI-LY1 were cultured in the same medium supplemented with 20% FBS. Media and supplements were purchased from Life Technologies except FBS, which was purchased from Sigma Chemical, Co. All cell lines were routinely screened for mycoplasma every month using MycoAlert (Lonza) and authentified using PCR-single-locus-technology (Eurofins). Cell lines were not grown more than 20 passages.

FL5.12 cells overexpressing BCL-2 or BCL-XL were kindly provided by Drs. A. Letai and L. Boise, respectively and cultured in RPMI 1640 supplemented with 10% Fetal Calf Serum, 2 mM L-glutamine and 10% of IL3-containing WEHI-3 cell culture supernatant. To assess sensitivity to BCL-2 inhibitors, FL5.12 cells were cultured for 24 h in the absence of IL-3 before treatment with BCL-2-inhibitors for an additional 24 h.

THP-1 knock-out BAX/BAK generation

For each round of transduction, lentiviral particles (Sigma Aldrich) were used to transduce 2 × 10^5 cells with a multiplicity of infection (MOI) of 10 for 2 h at 32° C in the presence of 8 µg/ml of polybrene. The virus-containing medium was then replaced with fresh medium and cells were incubated at 37° C for 48 h/72 h before starting selection.

THP-1 cells were first transduced with EF1a-Cas9-2A Neomycin (Sigma Aldrich). 96 h after transduction cells were put under neomycin selection (800 µg/ml, Invitrogen) for 10 days before being sorted as single-cells for the isolation of clones. One clone was then further transduced with pLV-U6g-EGFP-gRNA BAK1 (Sigma Aldrich, gRNA sequence: 5′ GCATGAAGTCGACCACGAAG 3′). 48 h after transduction cells were sorted as single-cells based on the expression of the GFP reporter gene. Cells were then further transduced with pLV-U6g-Puromycin gRNA BAX (Sigma Aldrich, gRNA sequence: 5′ CTGC AGGATGATTGCCGCCG 3′). 96 h after transduction cells were put under puromycin selection (1 µg/ml) for 9 days before being sorted as single-cells for the isolation of clones. BAX and BAK knock-out efficiency on different clones was verified by Western blot.

shRNA lentiviral transduction

Lentiviral particles containing Control shRNA (5′-CAACAAGATGAAGAGCACCAA-3′) or BAX shRNA (5′-GCCGGAACTGATCAGAACCAT-3) cloned into pcLV-U6-shRNA-EF1-eGFP-T2A-Puro) were purchased from Sirion Biotech (Martinsried, Germany). Lentiviral particles (1 × 10^7) were mixed with Polybrene at 8 µg/ml and transduced by spinonucleation for 1 h at 32° C. After 72 h, GFP positive-cells were sorted by FACS (SH800 Cell Sorter, Sony Biotechnology Inc.).

Immunoblotting

20 µg of whole cell lysates were analyzed by immunoblot using the following antibodies: MCL-1 (Santa Cruz S-19, sc-819), BAK (BD 556396), BAX (Santa Cruz sc-493), PARP (Cell Signaling 9542), Flag M2 (Sigma), BCL-XL (Transduction Lab 610212), BCL-2 (Santa Cruz sc-509), GAPDH (Cell Signaling 2118), caspase-3 and caspase-9 (kindly supplied by X. Sun, University of Leicester) and Tubulin (Calbiochem). BCL-2, BCL-XL, MCL-1, BAX and BAK were detected on independent blots. GAPDH detection was performed on each membrane as a loading control.

Co-immunoprecipitation

HeLa cells were transiently transfected, using Effecten reagent (Qiagen), with 3xFlag-tagged BCL-XL (accession number NM_138578) or BCL-2 (accession number NM_000633) expression vectors (p3xFlag-CMV10, Sigma). 24 h later, transfected Hela and RS4;11 cells were treated with S55746 or ABT-263 during 2 h and harvested in lysis buffer (10 mM Hepes pH 7.5, 150 mM KCl, 5 mM $MgCl_2$, 1 mM EDTA, 0.4% TritonX100), protease and phosphatase inhibitors cocktails (Calbiochem 539134 and 524625). HeLa cleared lysates were then subjected to immunoprecipitation with anti-Flag M2 agarose beads (Sigma) and RS4;11 cleared lysates were subjected to immunoprecipitation with anti-BCL-2 antibody. The immunoprecipitates and inputs were analyzed by immunoblot.

Cleaved PARP and cleaved caspase-3 immunodetection by MesoScale Discovery (MSD) assay

24 h after seeding, cells were treated with the indicated compounds for 4 h and harvested in lysis buffer (10 mM Hepes pH 7.4, 142.5 mM KCl, 5 mM $MgCl_2$, 1 mM EDTA, 1% NP40), protease and phosphatase inhibitors cocktails (Calbiochem 539134 and 524625)). Cleared lysates (5 µg) were assayed for immunodetection of cleaved PARP and cleaved caspase-3 with MSD Apoptosis Panel Whole Cell Lysate kit (MSD K15102D) in 96-well plates according to manufacturer's instructions, and analyzed on the Sector Image 2400. Control DMSO conditions were set to 100% and fold-activation calculated. For *ex vivo* experiments, once corrected with the blank, data from treated groups were represented as mean of the fold-increase compared to untreated control group.

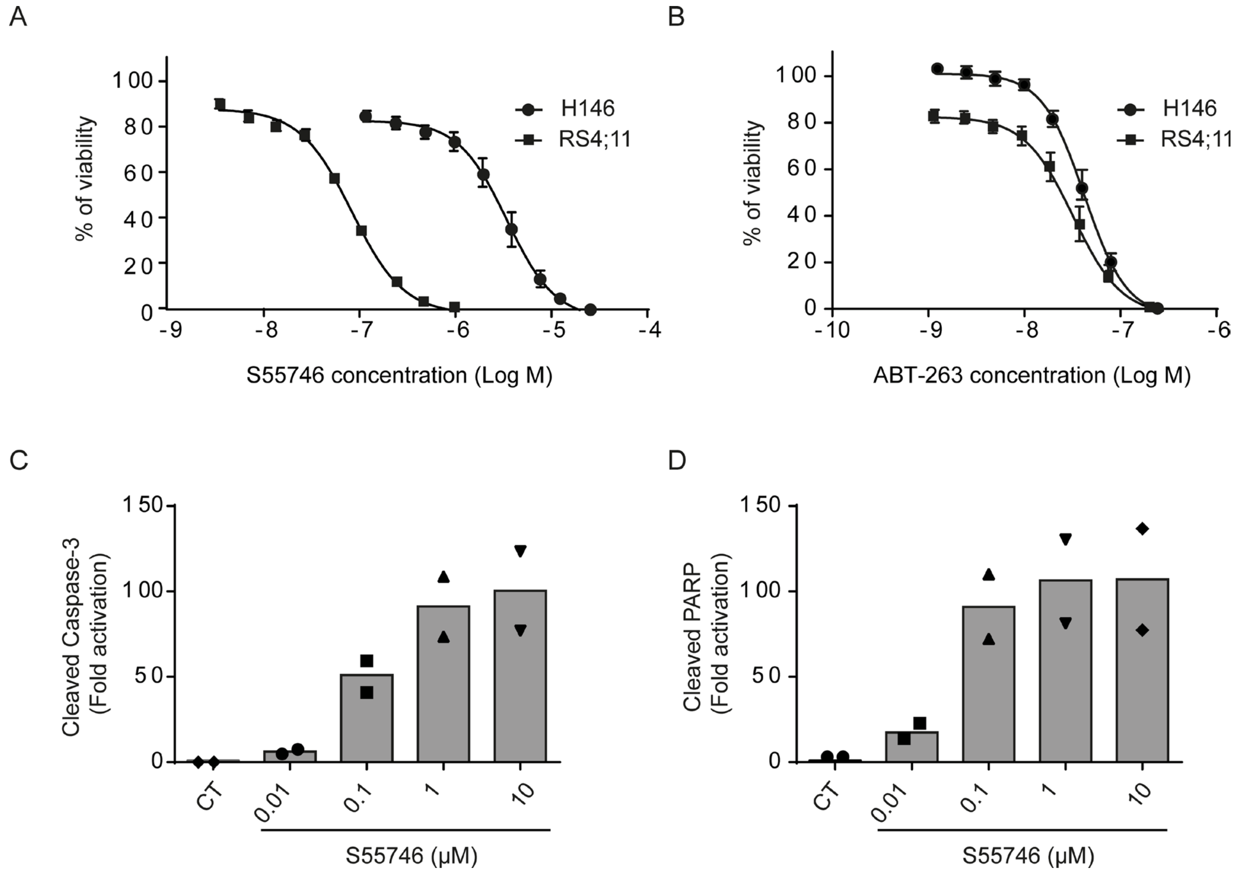

Supplementary Figure 1: S55746 induces disruption of the BCL-2/BAX complex and cell death in RS4;11 cell lines. (**A**) IC_{50} curves of S55746 in RS4;11 (squares) and H146 (circles). (**B**) IC_{50} curves of ABT-263 in RS4;11 (squares) and H146 (circles). For A and B: Curves are representative of 3 independent experiments. IC_{50} indicates concentration at which 50% of inhibition is reached. (**C** and **D**) RS4;11 cell lines were treated for 1h with increasing concentration of S55746. Cleaved caspase-3 (C) and cleaved PARP (D) was measured using MesoScale Discovery Apoptosis panel. Mean and individual data of 2 independent biological experiments are represented.

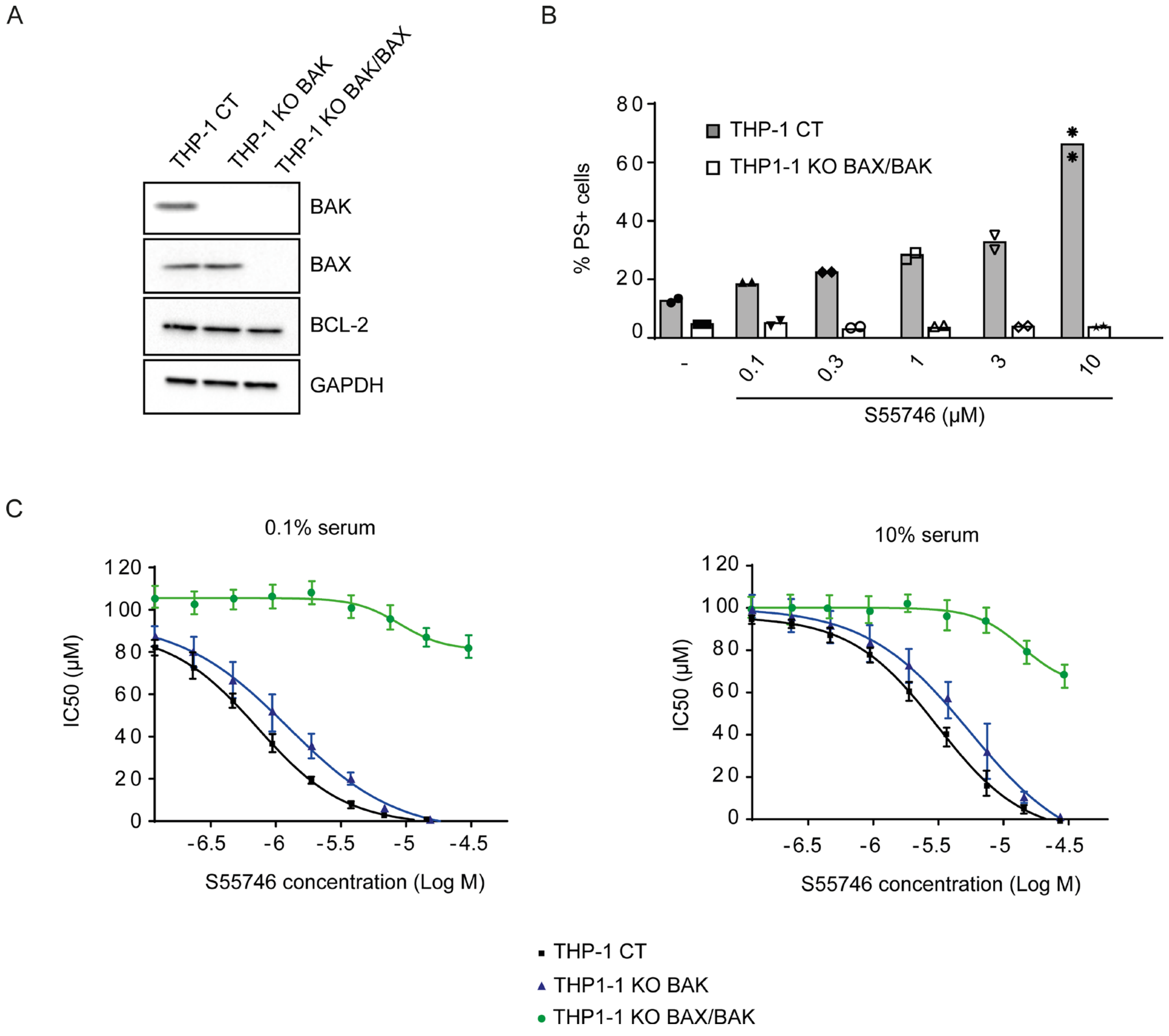

Supplementary Figure 2: S55746 induces apoptosis in a BAX/BAK dependent manner. (**A**) Expression of BCL-2, BAK and BAX was assessed by immunoblotting in THP-1 control (CT), THP1 knock-out BAK (KO BAK) or THP-1 knock-out BAX/BAK (KO BAX/BAK). GAPDH was used as a loading control. (**B**) Apoptosis induction in THP1 cells CT (control) or knock-out BAX/BAK treated with S55746 at the indicated concentration for 4 h. Cells were analyzed by flow cytometry for DAPI and annexin V-APC labeling. Mean and individual points from two biological replicates are shown. '–' indicates that cells were treated with DMSO only. (**C**) Viability of THP-1 control (CT) and knock-out for BAK (KO BAK) or BAX/BAK (KO BAX/BAK) either cultivated in 0.1% serum (left panel) or 10% serum (right panel) after incubation with increasing concentration of S55746 for 72 h. IC_{50} indicates concentration at which 50% of inhibition is reached. Mean and individual data of 3 independent experiments are shown.

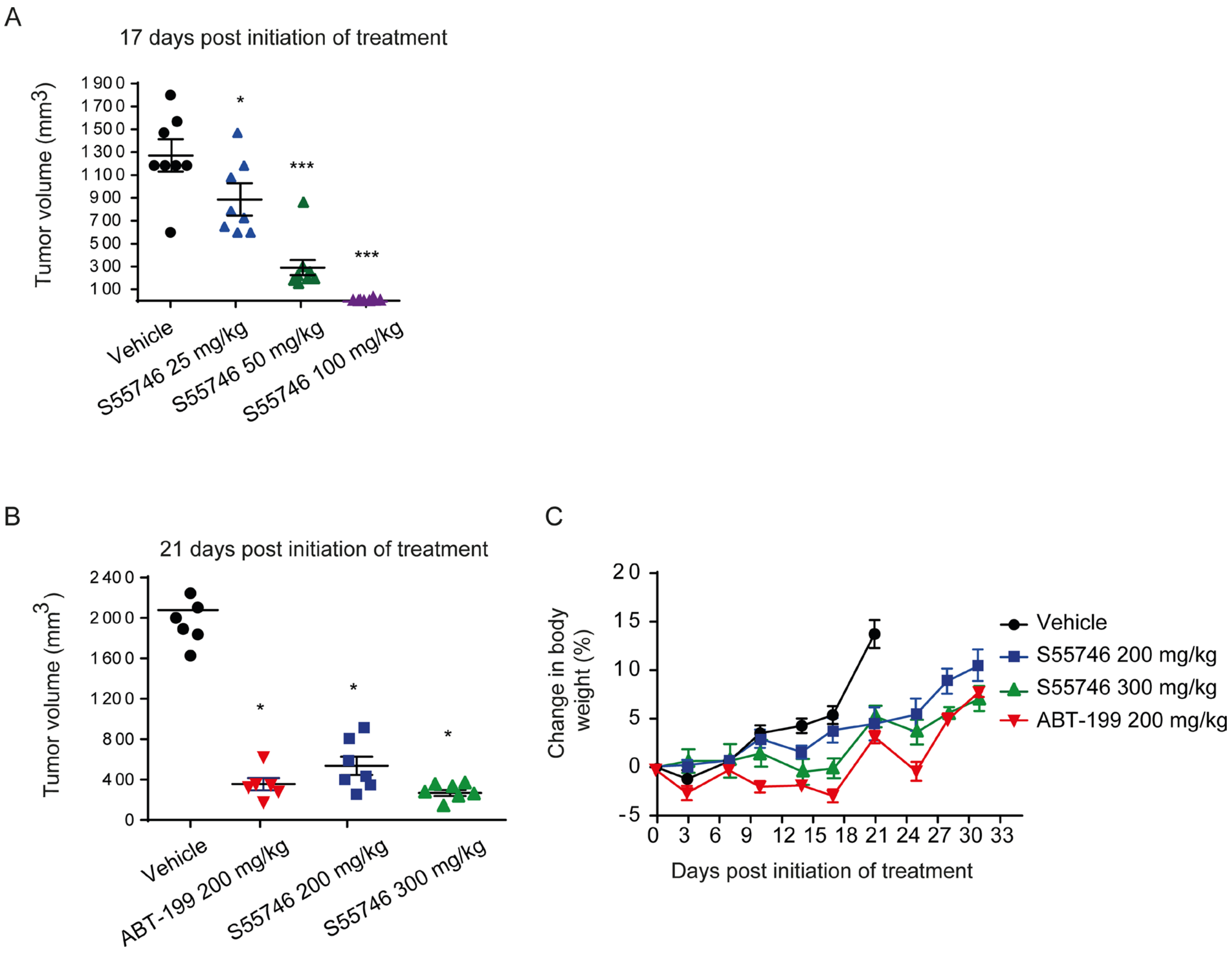

Supplementary Figure 3: Antitumor efficacy of S55746 in RS4;11 and Toledo xenograft models. Impact of treatment on body weight. (**A**) Mice were inoculated with RS4;11 cells and treated by oral gavage *per os* at 25 mg/kg (blue triangles), 50 mg/kg (green triangles) or 100 mg/kg (purple triangles) for 7 consecutive days after 27 days post-implantation. Individual data, 8 animals per treatment group are shown 17 days post beginning of the treatment. One way ANOVA on day 17, followed by Dunnett *post hoc* comparison was performed and always compared to vehicle, ($^{*}p < 0.05$; $^{***}p < 0.001$). (**B–C**) Mice were inoculated with Toledo cells and treated by oral gavage with ABT-199 at 200 mg/kg (inverted red triangles) and S55746 at 200 mg/kg (blue squares) or 300 mg/kg (green triangles) five days a week for 3 weeks 24 days after implantation. For B, individual data of 6–7 animals per treatment group are shown 47 days post-treatment. One way ANOVA on day 21 post beginning of the treatment, followed by Dunnett *post hoc* comparison was performed and always compared to vehicle, ($^{**}p < 0.01$; left panel). For C, percent change in body weight over time is shown for each group.

Supplementary Table 1: Primary Chronic Lymphocytic Leukemia cells freshly isolated from 7 patients were treated for 4 h with increasing concentrations of S55746

CLL number	p17 del	Spontaneous apoptosis at 4 h of culture (% of cells)	EC_{50} S55746 (nM)
**CLL1**	No	1	47.2
**CLL2**	No	3.7	13.8
**CLL3**	Yes	3.9	13.7
**CLL4**	No	5	12
**CLL5**	No	4.4	9.9
**CLL6**	Yes	8.2	7.8
**CLL7**	No	4	4.4

Individual EC50 of S55746 (half maximal effective concentration) of each CLL cells isolated from 7 patients are shown. Status of p17 deletion for each patient is indicated.

Supplementary Table 2: Primary Mantle Cell Lymphoma cells freshly isolated from 8 patients were treated for 24 h with increasing concentrations of S55746

Patient	EC50 S55746 (nM)
MCL1	110
MCL2	15
MCL3	12
MCL4	12
MCL5	5
MCL6	4
MCL7	3
MCL8	2.5

Cells were then analyzed by flow cytometry for PI, annexin V-FITC and CD19-APC positive labeling (top panel). Individual EC_{50} of S55746 (half maximal effective concentration) of each MCL cells isolated from 8 patients are shown.

Supplementary Table 3: Dose response anti-tumor effect of S55746 in RS4;11 xenograft model

Treatment	Day Post treatment	% T/C	Mean TV change (mm^3)
Vehicle (10 mL/kg)	17	100	1166.63 ± 120.37
S55746 (25 mg/kg)	17	67.1^{*}	782.38 ± 107.32
S55746 (50 mg/kg)	17	16.3^{***}	190.50 ± 76.72
S55746 (100 mg/kg)	17	-93.8^{***}	–98.13 ± 11.62

Results are expressed as means ± s.e.m. Legend: $^{*}p \leq 0.05$, $^{***}p \leq 0.001$; TV: Tumor Volume.

Supplementary Table 4: Anti-tumor effect of S55746 in Toledo xenograft model

Treatment	Day post treatment	% T/C	Mean TV change (mm^3)
Vehicle (10 mL/kg)	21	100	1837.91 ± 158.61
ABT-199 (200 mg/kg)	21	6^{*}	104.44 ± 70.31
S55746 (200 mg/kg)	21	13^{*}	239.03 ± 67.97
S55746 (300 mg/kg)	21	2^{*}	33.16 ± 37.38

Results are expressed as means +/- s.e.m. Legend: $^{*}\leq 0.05$; TV: Tumor Volume.
